# Revision of *Eudorylas* Aczél, 1940 (Diptera, Pipunculidae) in the Middle East, with the description of four new species

**DOI:** 10.3897/BDJ.8.e53609

**Published:** 2020-09-17

**Authors:** Behnam Motamedinia, Jeffrey Hunter Skevington, Scott Kelso

**Affiliations:** 1 Plant Protection Research Department, South Khorasan Agricultural and Natural Resources Research and Education Center, AREEO, Birjand, Iran Plant Protection Research Department, South Khorasan Agricultural and Natural Resources Research and Education Center, AREEO Birjand Iran; 2 Canadian National Collection of Insects, Arachnids and Nematodes, Agriculture and Agri-Food Canada, 960 Carling Avenue, ON K1A 0C6, Ottawa, Canada Canadian National Collection of Insects, Arachnids and Nematodes, Agriculture and Agri-Food Canada, 960 Carling Avenue, ON K1A 0C6 Ottawa Canada; 3 Carleton University, Biology Department, 207 Nesbitt Biology Building, 1125 Colonel By Drive, Ottawa, ON K1S 5B6, Ottawa, Canada Carleton University, Biology Department, 207 Nesbitt Biology Building, 1125 Colonel By Drive, Ottawa, ON K1S 5B6 Ottawa Canada

**Keywords:** big-headed flies, COI, diagnosis, distribution, DNA barcoding, Eudorylini, identification key, new species, west Palaearctic

## Abstract

**Background:**

The Middle Eastern species of *Eudorylas* Aczél, 1940 are revised through an integrative taxonomic approach by combining morphological and sequence data from the mitochondrial COI barcoding gene. Four new species of the genus *Eudorylas* are described, males and females of three species are associated, DNA sequence data of 11 Middle Eastern *Eudorylas* species are provided and 15 additional species are discussed. To facilitate their recognition, we provide diagnoses, descriptions, an identification key and distributional maps for all species.

**New information:**

The following new species are described from the Middle East: *E.avis* Motamedinia & Skevington **sp. n.**, *E.bihamatus* Motamedinia & Skevington **sp. n**., *E.corniculans* Motamedinia & Skevington **sp. n.**, *E.nasicus* Motamedinia & Skevington **sp. n.**

## Introduction

*Eudorylas* belongs to the tribe Eudorylini (Diptera, Pipunculidae) and is one of the most species-rich and cosmopolitan genera of Pipunculidae in the world with 416 valid species recognised (Skevington 2020). The first *Eudorylas* species was described in the 19th century as *Pipunculusfuscipes* (Zetterstedt, 1844). Aczél (1940) established *Eudorylas* and designated *Cephalopsopacus* Fallén, 1816 as the type species. Without studying type material, he transferred what he believed to be the relevant species from Becker (1897), Cresson (1911) and Sack (1935) to *Eudorylas*. The absence of propleural setae is one of the diagnostic characters of *Eudorylas* and overlooking or misinterpreting this caused serious instability in early attempts to place species in the genus. The original type species actually had a propleural fan of hairs and should never have been included in *Eudorylas*. Kuznetzov (1995) was the first to report on this and complicated things by synonymising *Eudorylas* with *Microcephalops* De Meyer, 1989 and introducing *Neodorylas* as a replacement name for *Eudorylas* (designating *Pipunculusfuscipes* Zetterstedt, 1844 as the type species). This action changed the generic name for over 30% of the world's pipunculids. De Meyer and Skevington (2001) appealed to the Commission of Zoological Nomenclature and their proposal to conserve the name *Eudorylas* by designating a new type species (*Pipunculusfuscipes* Zetterstedt, 1844) was accepted. *Neodorylas* became a junior objective synonym of *Eudorylas* following this and no generic upheaval occurred. The phylogenetic relationships of Eudorylini were studied by Skevington and Yeates (2001), who re-diagnosed the genus *Eudorylas* and proposed *Metadorylas* Rafael, 1987 as a synonym. Most recently, revision and description of several new species are provided in Australian (Skevington 2002a), Palaearctic and Oriental (Kehlmaier 2005a, Kehlmaier 2005b, Kehlmaier 2011), Afrotropical (Földvári 2003, Földvári 2013) regions.

The Eudorylas fauna in the Middle East (here defined to include Bahrain, Cyprus, Iran, Iraq, Israel, Jordan, Kuwait, Lebanon, Oman, Palestine, Qatar, Saudi Arabia, Syria, Turkey, United Arab Emirates and Yemen) currently comprises 15 species and now four are added, two of which (*E.bipertitus* Kehlmaier, 2005 and *E.flavicrus* De Meyer, 1995) are endemic (De Meyer 1995; Kehlmaier 2005b).

The aim of this study is to shed light on the species of *Eudorylas* occurring in the Middle East. To achieve this, we mainly focused on characters of male genitalia and sequence data obtained from the mitochondrial COI barcoding region. This paper presents information on taxonomy and includes distributional maps, diagnoses, photo illustrations of important morphological characters and an identification key for species occurring in the Middle East.

## Materials and methods

**Taxonomic description**

Male genitalia of *Eudorylas* provide the only absolute characters for secure species identification. Females have only been included in the type material when the DNA data match or geographic overlap with males is unequivocal. Genitalia preparations were made by separating the apical portion of the abdomen and heating in lactic acid (85%) at 100°C for 60–240 minutes and then moving the genitalia into a drop of glycerine on a microscope slide. Potassium hydroxide (KOH) was used for terminalia that were very darkly pigmented or that were to be used for photography. For this, terminalia were treated with 10% KOH at 100°C for 10–30 minutes then immersed in glacial acetic acid for 5 minutes to buffer the reaction and stop the clearing. Terminalia were then washed in ethanol before being placed in glycerine. Following clearing, dissection involved separating syntergosternite 8 and the epandrium from the remainder of the abdomen. After examination of the genitalia in glycerine, the dissections were transferred to microvials filled by glycerine and pinned below the specimen.

External characters were imaged using a Leica DFC450 module fitted on a Leica M205C stereomicroscope using a 0.6x lens. Final images were merged using the image-stacking software Zerene Stacker (Littlefield 2018). Images of the genitalia were taken using a Leica DM5500B microscope, equipped with a Leica DMC4500 module connected to a personal computer running the Leica Application Suite software (https://www.leica-microsystems.com), which includes an Auto-Montage module that combines multiple layers of photographs into a single fully-focused image. All photos were subsequently modified using Adobe Photoshop CS3^®^ imaging software for mounting photos in the plates.

Specimens examined are based on material deposited in the following collections: **CNC** = Canadian National Collection of Insects, Arachnids and Nematodes, Ottawa, Canada; **HMIM** = the Hayk Mirzayans Insect Museum, Tehran, Iran; **TAU** = Tel Aviv University, Israel. All specimens are labelled with a unique reference number from the CNC database (e.g. Jeff_Skevington_Specimen12345 or CNC_Diptera12345, abbreviated as JSS12345 and CNCD12345, respectively) and can be accessed at https://cnc.agr.gc.ca/. Species are presented in alphabetical order. SimpleMappr (Shorthouse 2010) was used to create the species distribution map. Morphological terminology used is based on Skevington (2002b) and Kehlmaier (2005a).

**Abbreviations**:

**LF:WF** = ratio of length of flagellum to its width.

**LW:MWW** = ratio of length of wing to maximum width of wing.

**LS:LTC** = ratio of length of pterostigma to length of third costal segment.

**LTC:LFC** = ratio of length of third costal segment to length of fourth costal segment.

**LT35:WT5** = ratio of length of tergites 3–5 to maximum width of tergite 5.

**WT5:LT5** = ratio of width of tergite 5 to its length.

**T5R:T5L** = ratio of length of right margin of tergite 5 to length of its left margin.

**LT35:WS8** = ratio of length of tergites 3–5 to width of syntergosternite 8.

**LS8:HS8** = ratio of length syntergosternite 8 to its height.

**MLE:MWE** = ratio of maximum length of epandrium to its maximum width (viewed dorsally).

**LP:LB** = ratio of length of piercer to length of base (viewed laterally).

**LDP:LPP** = ratio of length of distal part of piercer to length of its proximal part (viewed laterally).

**DNA extraction, PCR amplification and sequencing**

Total genomic DNA was extracted either from two legs or from whole specimens (dried or in alcohol) using the DNeasy Blood and Tissue Kit (Qiagen Inc., Santa Clara, CA, USA) following the manufacturer’s protocol. Following extraction, specimens were critical-point dried and deposited as vouchers in the CNC.

For DNA barcoding, a 658 bp fragment of the 5' end of the mitochondrial coding gene cytochrome oxidase subunit I (COI) was amplified using the primer pair LCO1490 and COI-Dipt-2183R (Gibson et al. 2011). In some cases, initial attempts to amplify the full COI barcode failed, presumably due to the degradation of the DNA. In these cases, a COI mini-barcode protocol was employed (Motamedinia et al. 2019) in order to amplify a 214 bp fragment (COI-Fx-C), located at the 3'-end of the COI barcode region, for species identification. In the case of putative new species, efforts were made to amplify the 5' and middle COI mini-barcode fragments (COI-Fx-A and COI-Fx-B, respectively) that, when combined, provide a complete COI barcode sequence. Oligonucleotides (primers) used in this study, are listed in Table 1. PCR amplifications were carried out in 25 μl volumes, including 15.7 μl ddH_2_O, 2.5 μl 10X Ex Taq PCR buffer (containing 20 mM MgCl_2_), 0.65 μl 25 mM MgCl_2_, 1 μl of each 10 μM primer, 2 μl 10 mM dNTPs, 0.15 μl Ex Taq HS DNA polymerase (TaKaRa Bio USA, Madison, WI, USA) and 2 μl total DNA. Amplification cycles were performed on an Eppendorf ep Gradient S Mastercycler (Eppendorf AG, Hamburg, Germany). All PCR and sequencing reactions were performed with the following thermal cycler conditions: 94°C for 3 mins x 1 cycle, 94°C for 45 secs, 45°C for 45 secs, 72°C for 1 min x 45 cycles, 72°C for 5 minutes x 1 cycle, followed by an unlimited step at 10°C. Amplification products were visualised on 1% agarose electrophoresis gels and purified prior to sequencing using either Clone-Well 0.8% Egels (Invitrogen™, Carlsbad, CA, USA) for full barcode amplicons or an ExoSAP-IT protocol (USB Corp., Cleveland, OH, USA) for COI-Fx amplicons. Sequencing reactions were carried out in 10 μl volumes, using the ABI BigDye Terminator v3.1 Cycle Sequencing kit (PE Applied Biosystems, Foster City, CA, USA). Bidirectional sequencing reactions were purified using the ABI ethanol/EDTA/sodium acetate precipitation protocol and analysed on an ABI 3500xl Genetic Analyzer (PE Applied Biosystems, Foster City, CA, USA). Sanger Sequencing was performed at CNC.

All sequence chromatograms were edited and contigs formed using Sequencher 5.4.6 (Gene Codes Corp., Ann Arbor, MI, USA). Resulting contigs were hand-aligned using Mesquite 3.6 (Maddison and Maddison 2018). Uncorrected pairwise genetic distances (p-distance) were calculated with Mega X (Kumar et al. 2018). Sequence accession numbers issued by GenBank (GB) are provided for each specimen.

## Taxon treatments

### Eudorylas

Aczél, 1940

ADF6C055-91CE-59A9-90EA-A9216693586F

Eudorylas *Pipunculusfuscipes*International Commission on Zoological Nomenclature 2002
Eudorylas **Synonyms**
Metadorylas Rafael, 1987 - Skevington and Yeates 2001: 438.
Neodorylas Kuznetzov, 1995 -International Commission on Zoological Nomenclature 2002.
Eudorylas Synonyms: MetadorylasSkevington and Yeates 2001: 438. Neodorylas: (ICZN 2002)

#### Diagnosis

Body length: 2.2-6.1 mm, wing length: 2.5-5.8 mm, pedicel with 3-7 upper and 1-5 lower bristles, flagellum grey to brownish-pruinose and tapering, frons often with a median keel, proepisternum with fan-like setal tuft, postprontal lobe with 3-10 setae along upper margin, scutellum with 10-20 short setae along posterior margin, hind tibiae with a wrinkled indentation, with or without erect anteromedial setae, pterostigma present, abdominal tergite 1 with 1-15 lateral bristles, syntergosternite 8 normal size and usually with membranous area, ejaculatory apodeme small and nail, fan or spade-shaped.

#### Distribution

Palaearctic (Andorra, Austria, Azerbaijan, Belgium, Bulgaria, China, Croatia, Cyprus, Czech Republic, Denmark, Egypt, Estonia, Finland, France, Japan, Georgia, Germany, Great Britain, Greece, Hungary, Iran, Ireland, Israel, Italy, Kazakhstan, Kyrgyz Republic, Latvia, Lithuania, Macedonia, Mongolia, Morocco, Netherlands, North Korea, Norway, Peru, Poland, Portugal, Romania, Russia, Slovakia, Slovenia, South Korea, Spain, Sri Lanka, Sweden, Switzerland, Tajikistan, Tunisia, Turkey, Turkmenistan, former Yugoslavia, Ukraine), Oriental (Borneo, India, Indonesia, Laos, Malaysia, Myanmar, Philippines, Singapore, Taiwan, Thailand, Vietnam), Afrotropical (Angola, Botswana, Burundi, Comoro Island, Congo, Gabon, Ghana, Kenya, Liberia, Madagascar, Malawi, Mozambique, Namibia, Rwanda, Tanzania, Trinidad, Uganda, Zimbabwe), Australian (Australia, Guam, New Zealand, Papua New Guinea), Nearctic (Canada, The United States of America) and Neotropical (Argentina, Bahamas, Brazil, Bolivia, Chile, Colombia, Costa Rica, Dominican Republic, Ecuador, El Salvador, Grenada, Jamaica, Mexico, Nicaragua, Panama, Paraguay) (Skevington 2020).

### Eudorylas auctus

Kehlmaier, 2005

739674C2-E8DB-5AE8-A8F6-E3FB44E3E15A

#### Diagnosis

This species can be recognised by the squared base of the surstyli, with inner finger-like projection in dorsal view; gonopods equal, inner side of basal half of hypandrium swollen in ventral view; phallus trifid and coiled twice; phallic guide straight in lateral view (for illustrations, see Kehlmaier 2005a: Figure 47a, l).

#### Distribution

**Figure 1.**
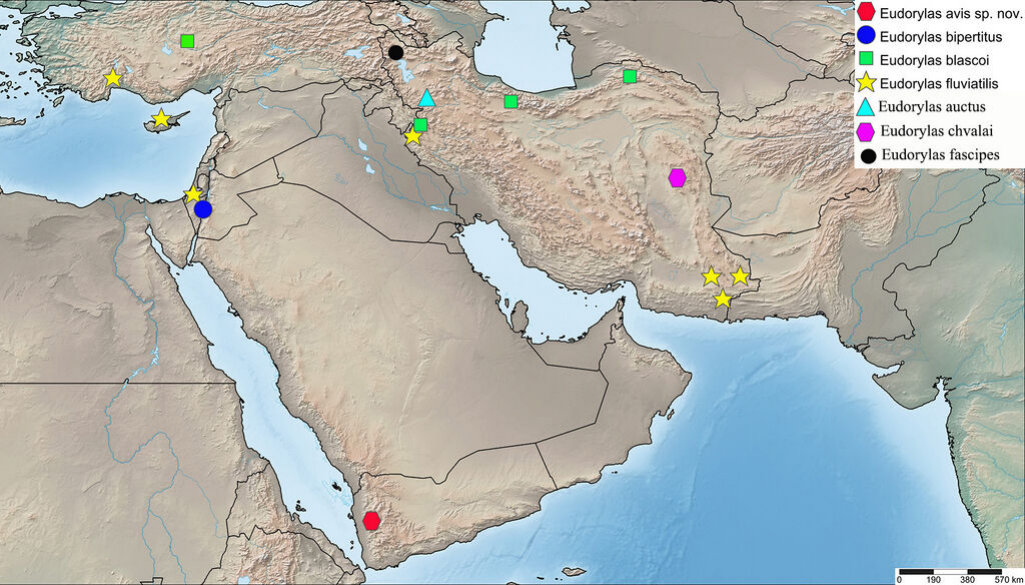
*Eudorylas* species distribution in the Middle East.

England, Germany, Greece, Iran (Fig. 1), Italy, Kyrgyz Republic, Spain, Tajikistan, Uzbekistan (Kehlmaier 2005a, Kazerani et al. 2017, Skevington 2020).

#### Notes

Our single DNA barcode of *Eudorylasauctus* from Germany (JSS15405) overlaps with barcodes of *E.obscurus* from France (CNC464954) and *E.longifrons* from Iran (GB: LT671752). The genitalia of these species are different, so this is likely just a case of incomplete lineage sorting due to ancestral hybridisation or the fact that these are young species whose barcodes have not yet diverged, as seen in many other taxa (e.g. Skevington 2005, Skevington et al. 2007, Young et al. 2016). It does raise the possibility that these three taxa are part of a single variable species with polymorphic genitalia. Future work should explore population genetics within this cluster of species, perhaps using the rapidly evolving marker, ITS2.

### Eudorylas avis

Motamedinia & Skevington, 2020 sp. n.

2490EA18-FD60-5BFE-A244-C2CB17592186

318851F6-56DB-4EE6-B73B-0CE034798FC2

#### Materials

- **Type status:** Holotype. **Occurrence:** catalogNumber: CNCD6829; individualCount: 1; sex: male; lifeStage: adult; associatedSequences: GB: MN549658; **Taxon:** scientificName: *Eudorylasavis*; **Location:** country: Yemen; locality: 12 km NW of Manakhah; decimalLatitude: 15.071944; decimalLongitude: 43.740833; **Event:** samplingProtocol: Malaise trap; eventDate: 2003-06-24/08-04; **Record Level:** institutionCode: CNC

#### Description

**Figure 2.**
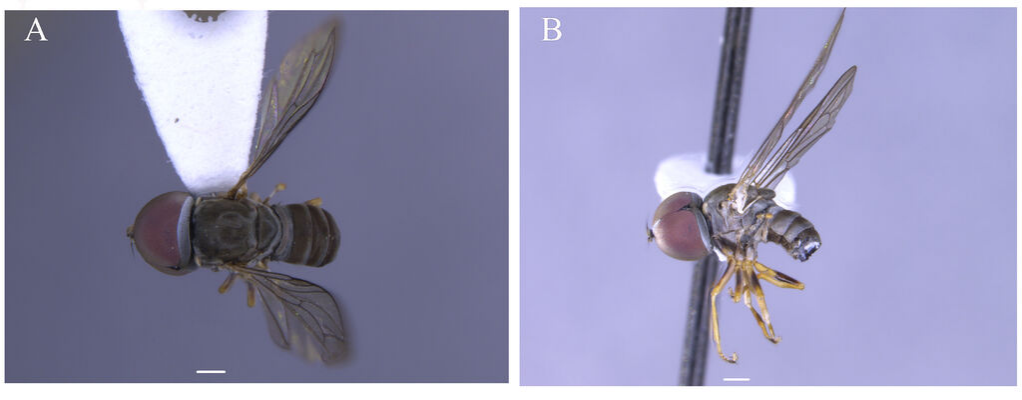
Male of *Eudorylasavis* Motamedinia & Skevington sp. n. (CNCD6829) (A) habitus in dorsal view (terminalia removed); (B) habitus in lateral view. Scale bar = 0.5 mm.

**Figure 3.**
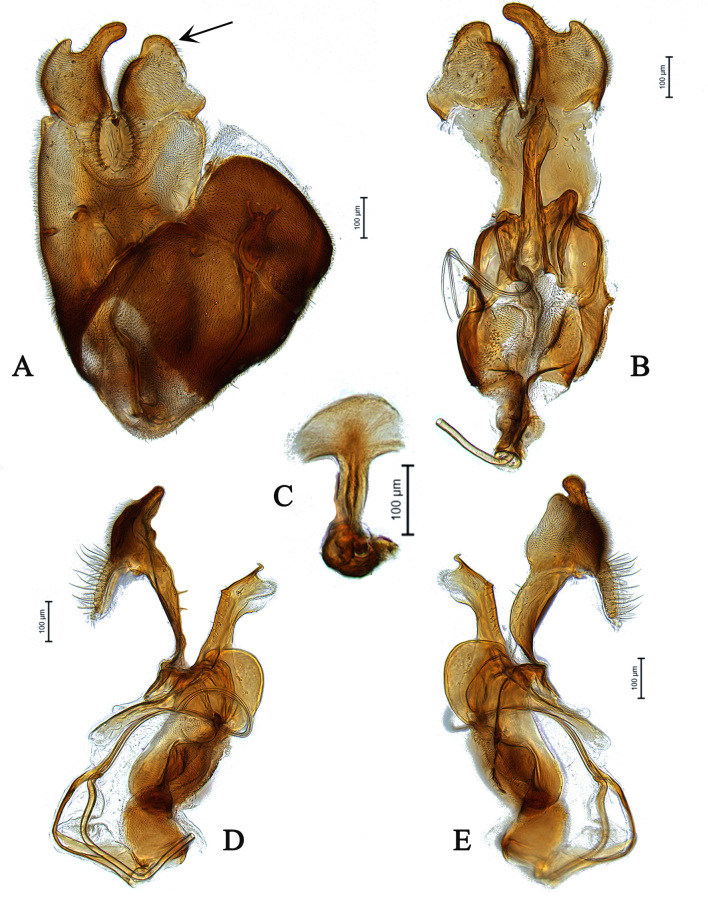
Male genitalia of *Eudorylasavis* Motamedinia & Skevington sp. n. (CNCD6829) (A) in dorsal view; (B) in ventral view; (C) ejaculatory apodeme; (D, E) in lateral view.

**Male** (Fig. 2A, B). Body length (excluding antennae): 3.4 mm. **Head**. Face grey pruinose. Scape dark with one short upper bristle, pedicel dark with three short upper bristles and three short lower bristles, flagellum and base of arista light brown; flagellum tapering and grey-white pruinose (LF:WF = 2.5). Labellum yellow. Eyes meeting for a distance of 12 facets. Frons silver-grey pruinose. Vertex black, lacking pruinosity. Occiput dark and grey pruinose. **Thorax**. Postpronotal lobe light yellow, grey pruinose with 6-7 short bristles along upper margin (up to 0.05 mm). Prescutum black, grey pruinose. Scutum black, brown pruinose with scattered long setae at anterior supra-alar area. Scutellum black, brown pruinose with eight dark setae along posterior margin (up to 0.4 mm). Subscutellum dark, grey pruinose. Pleura dark brown, grey pruinose. **Wing**. Length: 4.5 mm. LW:MWW = 3.0. Wing almost entirely covered with microtrichia. Pterostigma dark-brown and complete. LS:LTC = 1.0. LTC:LFC = 1.3. Cross-vein r-m reaches dm shortly after one-third of the cell's length. Halter length: 0.5 mm. Light brown. **Legs**. Coxae dark, grey pruinose. Mid coxa with four black anterior bristles. Trochanters light brown, partly grey pruinose. Femora dark with light brown apices and light brown posteriorly. Mid and hind femora bearing two rows of dark, peg-like anteroventral spines in apical one third. Tibiae light brown, grey pruinose, with two rows of short setae on anterior and three rows on posterior side. Hind tibiae bearing one or two wrinkled indentations in middle. Tarsi yellowish but distitarsi dark, with scattered dark setae at anterior margin. Claws yellow with black tips. **Abdomen**. Ground colour dark brown, tergites 1 black, grey pruinose, with one long and 5-6 short lateral bristles. Tergites 2–4 laterally grey pollinose extending a little on to dorsal surface along posterior margin, otherwise brown pollinose. Sternites dark brown, brown pruinose. Syntergosternite 8 dark, dark pruinose. Membranous area large, almost reaching epandrium, vertically directed. **Genitalia**. Genital capsule in dorsal view: epandrium and surstyli brown, brown pruinose. Epandrium longer than wide (MLE:MWE = 1.3) (Fig. 3A). Surstyli asymmetrical, right larger than left one. Left surstylus rather rectangular-shaped. Base of left surstylus wider than the right one. Right surstylus with an inner finger-like projection curved towards left surstylus, left surstylus with a projection at apex (Fig. 3A). Genital capsule in ventral view: gonopods unequal, right is longer than the left one (Fig. 3B). Genital capsule in lateral view: both surstyli with a finger-like projection at apices, right surstylus with shorter finger-like projection than left one, base of right surstylus broader than left one (Fig. 3D, E). Phallus trifid; phallic guide strongly broadened, bent shortly before apex with two ventrally feather-like projections and apically with a small projecting hook (Fig. 3D, E); hypandrial apodeme extended (Fig. 3D, E). Ejaculatory apodeme spade-shaped (Fig. 3C).

#### Diagnosis

This species can be distinguished by the shape of the surstyli in dorsal view; base of left surstylus broader than the right one, right surstylus apically with inner long finger-like projection curved towards left one and small outer finger-like projection (Fig. 3A); large membranous area (Fig. 3A); phallic guide bent before apex with two feather-like projections in lateral view (Fig. 3D, E); distinct hypandrial apodeme in lateral view (Fig. 3D, E).

#### Etymology

The specific epithet is derived from the Latin avis which means bird and refers to the similarity between the shape of the phallic guide apically in lateral view to that of a bird.

#### Distribution

Yemen (Fig. 1).

#### Notes

Based on DNA barcodes, this species is closest to one or more species from South Africa (2.09-2.45% pairwise divergence). Unidentified female specimens from Yemen (CNCD6818) and Angola (CNC395962) are sufficiently different that they are not likely the same species (4.21% and 3.14%, respectively).

### Eudorylas bihamatus

Motamedinia & Skevington, 2020 sp. n.

6E956838-2766-541D-A3C4-50BE5A2293B3

ED88C435-14F2-4108-AFAF-C4897EEF5CCE

#### Materials

- **Type status:** Holotype. **Occurrence:** catalogNumber: JSS52191; recordedBy: M. Parchami-Araghi; individualCount: 1; sex: male; lifeStage: adult; associatedSequences: GB: MN549663; **Taxon:** scientificName: *Eudorylasbihamatus*; **Location:** country: Iran; stateProvince: Khuzestan; locality: Shush; decimalLatitude: 32.1; decimalLongitude: 48.433333; **Event:** samplingProtocol: Malaise trap; eventDate: 2015-02-11/05-10; **Record Level:** institutionCode: CNC
- **Type status:** Paratype. **Occurrence:** catalogNumber: JSS52313; recordedBy: M. Parchami-Araghi; individualCount: 1; sex: male; lifeStage: adult; **Taxon:** scientificName: *Eudorylasbihamatus*; **Location:** country: Iran; stateProvince: Khuzestan; locality: Shush; decimalLatitude: 32.1; decimalLongitude: 48.433333; **Event:** samplingProtocol: Malaise trap; eventDate: 2015-02-11/05-10; **Record Level:** institutionCode: CNC
- **Type status:** Paratype. **Occurrence:** catalogNumber: JSS52314; recordedBy: M. Parchami-Araghi; individualCount: 1; sex: male; lifeStage: adult; **Taxon:** scientificName: *Eudorylasbihamatus*; **Location:** country: Iran; stateProvince: Khuzestan; locality: Shush; decimalLatitude: 32.1; decimalLongitude: 48.433333; **Event:** samplingProtocol: Malaise trap; eventDate: 2015-02-11/05-11; **Record Level:** institutionCode: CNC
- **Type status:** Paratype. **Occurrence:** catalogNumber: JSS52315; recordedBy: M. Parchami-Araghi; individualCount: 1; sex: male; lifeStage: adult; **Taxon:** scientificName: *Eudorylasbihamatus*; **Location:** country: Iran; stateProvince: Khuzestan; locality: Shush; decimalLatitude: 32.1; decimalLongitude: 48.433333; **Event:** samplingProtocol: Malaise trap; eventDate: 2015-02-11/05-10; **Record Level:** institutionCode: CNC
- **Type status:** Paratype. **Occurrence:** catalogNumber: JSS52316; recordedBy: M. Parchami-Araghi; individualCount: 1; sex: male; lifeStage: adult; **Taxon:** scientificName: *Eudorylasbihamatus*; **Location:** country: Iran; stateProvince: Khuzestan; locality: Shush; decimalLatitude: 32.1; decimalLongitude: 48.433333; **Event:** samplingProtocol: Malaise trap; eventDate: 2015-02-11/05-10; **Record Level:** institutionCode: CNC
- **Type status:** Paratype. **Occurrence:** catalogNumber: JSS52317; recordedBy: M. Parchami-Araghi; individualCount: 1; sex: male; lifeStage: adult; **Taxon:** scientificName: *Eudorylasbihamatus*; **Location:** country: Iran; stateProvince: Khuzestan; locality: Shush; decimalLatitude: 32.1; decimalLongitude: 48.433333; **Event:** samplingProtocol: Malaise trap; eventDate: 2015-02-11/05-10; **Record Level:** institutionCode: HMIM

#### Description

**Figure 4.**
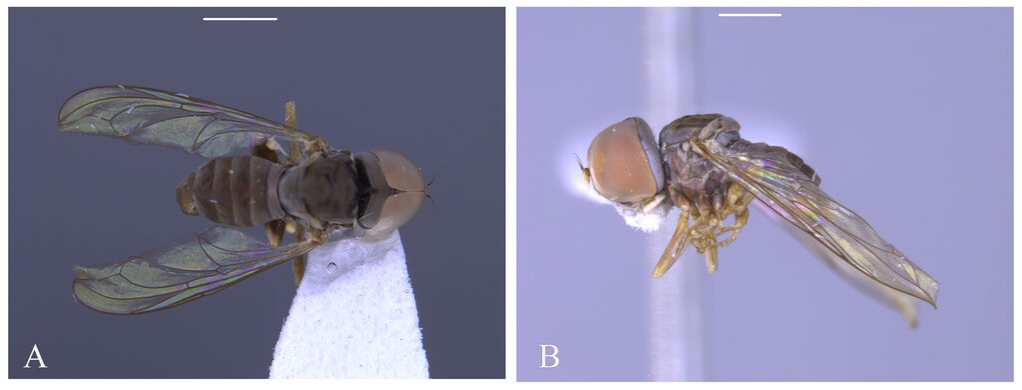
Male of *Eudorylasbihamatus* Motamedinia & Skevington sp. n. (JSS52315) (A) habitus in dorsal view; (B) habitus in lateral view. Scale bar = 1 mm.

**Figure 5.**
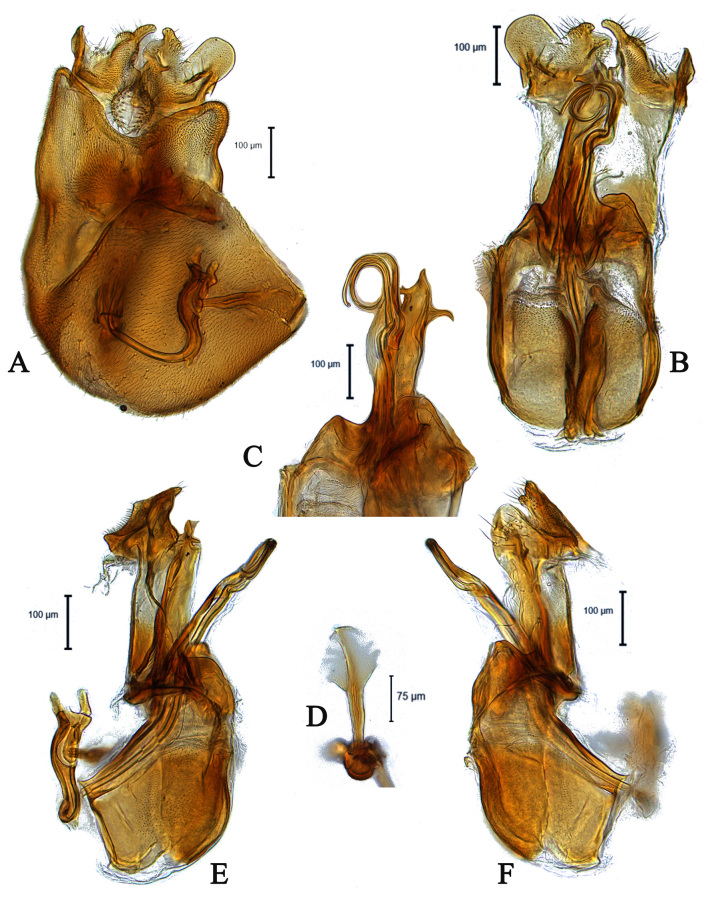
Male genitalia of *Eudorylasbihamatus* Motamedinia & Skevington sp. n. (JSS52191) (A) in dorsal view; (B) in ventral view; (C) phallus and phallic guide in ventrolateral view; (D) ejaculatory apodeme; (E, F) in lateral view.

**Male** (Fig. 4 A, B). Body length (excluding antennae): 3.8-4.1 mm (n = 6). **Head.** Scape black, pedicel and arista dark brown, pedicel with a pair of short upper bristles, flagellum light brown, tapering and grey pruinose (LF:WF = 3.0); arista with thickened base. Eyes meeting for a distance of 12-13 facets. Frons dark silver-grey pruinose. Vertex black, bearing an elevated slightly ocellar triangle. Occiput dark and grey pruinose. **Thorax.** Postpronotal lobe brown, grey pruinose with 2-3 short bristles along upper margin. Prescutum and scutum black with scattered long setae at anterior supra-alar area. Scutellum black with eight thin short setae along posterior margin (up to 0.04 mm). Subscutellum black, grey pruinose. Pleura brown. Wing. Length: 3.5–3.6 mm. LW:MWW = 3.1. Wing almost entirely covered with microtrichia. Pterostigma brown and complete. LS:LTC = 1.0. LTC:LFC = 1.0. Cross-vein r-m reaches dm shortly after one-third of the cell’s length. Halter length: 0.5 mm, base dark brown stem and knob light brown. **Legs.** dark brown, grey pruinose. Mid coxa with three brown anterior bristles. Trochanters partly grey pruinose. Femora dark brown with pale apices, grey pruinose. Mid and hind femora bearing two rows of dark anteroventral small spines in apical half. Tibiae grey pruinose, with two rows of short setae on anterior side and three rows on posterior. Hind tibia with three wrinkled indentations in middle without erect anteromedial setae. Tarsi yellowish with light brown bristles at posterior margin and dark brown scattered setae at anterior margin. Pulvilli light brown, slightly large. Claws light brown with black tips. **Abdomen.** Ground colour dark brown, tergite 1 silver-grey pruinose, with three dark lateral bristles (up to 0.1 mm). Tergites 2–5 posterolaterally grey pruinose, slightly extending on to dorsal surface along posterior margin, extending on to dorsal surface, otherwise brown pruinose. Tergite 5 slightly wider than other tergites and almost asymmetrical in dorsal view (LT35:WT5 = 1.2, WT5:LT5 = 0.4, T5R:T5L = 1.2). Sternites brown, lighter than tergites, grey pruinose. Syntergosternite 8 enlarged, dark brown and grey pruinose. Viewed laterally, longer than high (LS8:HS8 = 1.8). Membranous area large and triangular-shaped caudally. **Genitalia**. Genital capsule in dorsal view: epandrium and surstyli brown. Epandrium longer than wide (MLE:MWE = 1.1). Surstyli rather asymmetrical, wider than long, both surstyli with a small inner finger-like projections, tip of both finger-like projections curved towards inner side, left surstylus with a broad projection at the base (Fig. 5A). Genital capsule in ventral view: subepandrial sclerite wide without setae (Fig. 5B); gonopods unequal, right is higher and broader than left one (Fig. 5B); phallic guide strong and straight, pointed apically, lateraly with two downwards sclerotised hook-like projections shortly before the apex, right hook is longer than left one (Fig. 5C). Phallus trifid with circular ejaculatory ducts (Fig. 5B, C). Genital capsule in lateral view: both surstyli wide at the base with a small finger-like projection at apex (Fig. 5E, F). Ejaculatory apodeme small, spade-shaped (Fig. 5D).

#### Diagnosis

This species can be recognised by the shape of surstyli in dorsal view, wider than long, both surstyli with a small inner finger-like projection apically, left surstylus with a broad projection at outer side (Fig. 5A); phallic guide strong and straight with two laterally hook-like projections shortly before the apex (Fig. 5C).

#### Etymology

The specific name is derived from the Latin bihamatus which means "with two hooks" and references the two lateral hooks on its phallic guide.

#### Distribution

Iran (Fig. 6).

#### Notes

Based on DNA barcodes, *Eudorylasbihamatus* sp. n. is genetically most similar to *E.corniculans* sp. n. (5.26% pairwise divergence).

### Eudorylas bipertitus

Kehlmaier, 2005

FB269D11-8DE7-5EC9-9BB8-F071FDF269BE

#### Materials

- **Type status:** Other material. **Occurrence:** catalogNumber: JSS50815; recordedBy: M. E. Irwin; individualCount: 1; sex: male; lifeStage: adult; associatedSequences: GB: MN549653; **Taxon:** scientificName: *Eudorylasbipertitus*; **Location:** country: Israel; locality: Arava Valley, nr Hazeva, Shizaf Nature Res. side channel of Wadi Shahak; decimalLatitude: 30.75; decimalLongitude: 35.25; **Event:** samplingProtocol: Malaise trap; eventDate: 1995-04-06; **Record Level:** institutionCode: CNC
- **Type status:** Other material. **Occurrence:** catalogNumber: JSS50816; recordedBy: M. E. Irwin; individualCount: 1; sex: male; lifeStage: adult; **Taxon:** scientificName: *Eudorylasbipertitus*; **Location:** country: Israel; locality: Arava Valley, nr Hazeva, Shizaf Nature Res. side channel of Wadi Shahak; decimalLatitude: 30.75; decimalLongitude: 35.25; **Event:** samplingProtocol: Malaise trap; eventDate: 1995-03-14; **Record Level:** institutionCode: TAU
- **Type status:** Other material. **Occurrence:** catalogNumber: JSS50817; recordedBy: M. E. Irwin; individualCount: 1; sex: male; lifeStage: adult; **Taxon:** scientificName: *Eudorylasbipertitus*; **Location:** country: Israel; locality: Arava Valley, nr Hazeva, Shizaf Nature Res. side channel of Wadi Shahak; decimalLatitude: 30.75; decimalLongitude: 35.25; **Event:** samplingProtocol: Malaise trap; eventDate: 1995-03-15; **Record Level:** institutionCode: TAU
- **Type status:** Other material. **Occurrence:** catalogNumber: JSS50818; recordedBy: M. E. Irwin; individualCount: 1; sex: male; lifeStage: adult; **Taxon:** scientificName: *Eudorylasbipertitus*; **Location:** country: Israel; locality: Arava Valley, nr Hazeva, Shizaf Nature Res. side channel of Wadi Shahak; decimalLatitude: 30.75; decimalLongitude: 35.25; **Event:** samplingProtocol: Malaise trap; eventDate: 1995-03-22; **Record Level:** institutionCode: TAU

#### Diagnosis

This species can be recognised by the divided phallic guide in ventral view, separated directly from gonopods, left is stronger and wider than right one (Fig. 7C); both surstyli with a wide base and finger-like projection apically, left towards right one in dorsal view (Fig. 7A, B); gonopods asymmetrical (Fig. 7C); ejaculatory apodeme spade-shaped (Fig. 7D).

#### Distribution

India (unpublished data, CNC485558 and CNC485558), Israel (Kehlmaier 2005b) (Fig. 1).

#### Notes

Based on DNA barcodes, *Eudorylasbipertitus* is genetically most similar to *E.corniculans* sp. n. and *E.bihamatus* sp. n. (7.74% and 8.57% pairwise divergence, respectively) and to two unnamed South African *Eudorylas* species (8.51% pairwise divergence).

### Eudorylas blascoi

De Meyer, 1997

19760F86-6675-5F3E-A88F-4ABA3C655072

#### Materials

- **Type status:** Other material. **Occurrence:** catalogNumber: JSS52179; recordedBy: M. Zardouei; individualCount: 1; sex: male; lifeStage: adult; associatedSequences: GB: MN549665; **Taxon:** scientificName: *Eudorylasblascoi*; **Location:** country: Iran; stateProvince: Kermanshah; locality: Dodan; decimalLatitude: 35.10; decimalLongitude: 46.20; **Event:** samplingProtocol: funnel Malaise trap; eventDate: 2016-05-20; **Record Level:** institutionCode: CNC
- **Type status:** Other material. **Occurrence:** catalogNumber: JSS52039; recordedBy: B. Motamedinia; individualCount: 1; sex: female; lifeStage: adult; associatedSequences: GB: MN549654; **Taxon:** scientificName: *Eudorylasblascoi*; **Location:** country: Iran; stateProvince: North Khorasan; locality: Darkesh; decimalLatitude: 37.433333; decimalLongitude: 56.733333; **Event:** samplingProtocol: sweeping; eventDate: 2016-07-23; **Record Level:** institutionCode: CNC
- **Type status:** Other material. **Occurrence:** catalogNumber: JSS52023; recordedBy: B. Motamedinia; individualCount: 1; sex: female; lifeStage: adult; associatedSequences: GB: MN549646; **Taxon:** scientificName: *Eudorylasblascoi*; **Location:** country: Iran; stateProvince: North Khorasan; locality: Biar; decimalLatitude: 37.5395; decimalLongitude: 56.943333; **Event:** samplingProtocol: Malaise trap; eventDate: 2016-06-10/24; **Record Level:** institutionCode: CNC
- **Type status:** Other material. **Occurrence:** catalogNumber: JSS52223; recordedBy: A. Jabari; individualCount: 1; sex: female; lifeStage: adult; associatedSequences: GB: MN549648; **Taxon:** scientificName: *Eudorylasblascoi*; **Location:** country: Iran; stateProvince: Alborz; locality: Taleghan; decimalLatitude: 36.166667; decimalLongitude: 50.75; **Event:** samplingProtocol: Malaise trap; **Record Level:** institutionCode: CNC

#### Diagnosis

The male of this species can be recognised by a large membranous area, as wide as long; unequal gonopods (Fig. 8B); phallic guide thin in basal half and broad in apical half, with the hook-like projection in the middle pointing upwards in ventral view (Fig. 8B); right surstylus with a strong projection in the middle of inner margin in dorsal view (Fig. 8A).

#### Distribution

France, Greece, Iran, Italy, Portugal, Spain, Turkey, Uzbekistan (Kehlmaier 2005a, Motamedinia et al. 2017, Kehlmaier et al. 2019, Skevington 2020) (Fig. 1).

#### Notes

*Eudorylasblascoi* is part of the *E.mutillatus* species complex. Based on uncorrected pairwise genetic distances (p-distance), *E.blascoi* differs from *E.mutillatus* by 3.58%.

### Eudorylas chvalai

Kozánek, 1988

76F096F1-9D1B-5EF5-98C7-E701F78C9405

#### Diagnosis

This species can be recognised by asymmetrical surstyli, left surstylus slightly triangular-shaped, right surstylus quadratic-shaped at the base with long inner finger-like projection and a short outer one in dorsal view; gonopods unequal, right larger than left one in ventral view; phallic guide straight, but at dorsal margin, concave in apical two thirds in lateral view (for illustration, see Kehlmaier 2005a: Figure 69a, k).

#### Distribution

Greece, Iran (Fig. 1), Turkmenia (Kehlmaier 2005a, Motamedinia et al. 2017, Skevington 2020).

#### Notes

*Eudorylaschvalai* is not very similar genetically to any other *Eudorylas* species. Using the BOLD DNA identification engine (The Barcode of Life Data System 2020), the most similar recorded DNA barcode, a specimen from Pakistan on BOLD, is only 91.5% similar. *Eudorylasblascoi* is 89.97-90.13% similar.

### Eudorylas corniculans

Motamedinia & Skevington, 2020 sp. n.

DD4D7E80-AC64-5D15-85C3-10567260660A

668125A7-9606-4E17-ACDD-E0C556CCE14F

#### Materials

- **Type status:** Holotype. **Occurrence:** catalogNumber: JSS52187; recordedBy: M. Zardouei; individualCount: 1; sex: male; lifeStage: adult; associatedSequences: GB: MN549644; **Taxon:** scientificName: *Eudorylascorniculans*; **Location:** country: Iran; stateProvince: Kermanshah; locality: Sarpolezahab; decimalLatitude: 34.466667; decimalLongitude: 45.816667; **Event:** samplingProtocol: Malaise trap; eventDate: 2016-05-14; **Record Level:** institutionCode: CNC
- **Type status:** Paratype. **Occurrence:** catalogNumber: JSS52206; recordedBy: E. Gilasian; individualCount: 1; sex: female; lifeStage: adult; associatedSequences: GB: MN549645; **Taxon:** scientificName: *Eudorylascorniculans*; **Location:** country: Iran; stateProvince: Khuzestan; locality: Shush; decimalLatitude: 32.1; decimalLongitude: 48.433333; **Event:** samplingProtocol: Malaise trap; eventDate: 2015-03-11/05-10; **Record Level:** institutionCode: CNC
- **Type status:** Paratype. **Occurrence:** catalogNumber: JSS52237; recordedBy: E. Gilasian; individualCount: 1; sex: female; lifeStage: adult; associatedSequences: GB: MN549655; **Taxon:** scientificName: *Eudorylascorniculans*; **Location:** country: Iran; stateProvince: Khuzestan; locality: Shush; decimalLatitude: 32.1; decimalLongitude: 48.433333; **Event:** samplingProtocol: Malaise trap; eventDate: 2015-03-11/05-10; **Record Level:** institutionCode: CNC
- **Type status:** Paratype. **Occurrence:** catalogNumber: JSS52312; recordedBy: M. Parchami-Araghi; individualCount: 1; sex: male; lifeStage: adult; **Taxon:** scientificName: *Eudorylascorniculans*; **Location:** country: Iran; stateProvince: Khuzestan; locality: Shush; decimalLatitude: 32.1; decimalLongitude: 48.433333; **Event:** samplingProtocol: Malaise trap; eventDate: 2013-06-29/07-04; **Record Level:** institutionCode: HMIM
- **Type status:** Paratype. **Occurrence:** catalogNumber: JSS50776; recordedBy: A. Maklakov; individualCount: 1; sex: male; lifeStage: adult; **Taxon:** scientificName: *Eudorylascorniculans*; **Location:** country: Israel; locality: Nahal Shezaf; decimalLatitude: 30.716667; decimalLongitude: 35.266667; **Event:** samplingProtocol: Malaise trap; eventDate: 1997-11-30; **Record Level:** institutionCode: TAU
- **Type status:** Paratype. **Occurrence:** catalogNumber: JSS50785; recordedBy: A. Maklakov; individualCount: 1; sex: male; lifeStage: adult; **Taxon:** scientificName: Eudorylas *corniculans*; **Location:** country: Israel; locality: Nahal Shahaq; decimalLatitude: 30.733333; decimalLongitude: 35.233333; **Event:** samplingProtocol: Malaise trap; eventDate: 1997-07-01; **Record Level:** institutionCode: TAU

#### Description

**Male** (Fig. 9A, B). Body length (excluding antennae): 3.2–3.3 mm (n = 2). **Head.** Scape, pedicel and arista dark brown, pedicel with a pair of short upper and lower bristles, lower bristles as long as upper bristles, flagellum tapering and light brown pruinose (LF:WF = 3.0); arista with thickened base. Eyes meeting for a distance of 8-9 facets. Frons dark silver-grey pruinose. Vertex black, bearing an elevated slightly ocellar triangle. Occiput dark and grey and brown pruinose with a row of long setae along posterior margin. **Thorax.** Postpronotal lobe light brown, grey pruinose and with four to five postpronotal light brown bristles along upper margin (up to 0.05 mm). Prescutum and scutum black, predominantly grey-brown pruinose, with two uniseriate dorsocentral rows of dark bristles and longer supra-alar bristles. Scutellum black with 8 thin short setae along posterior margin (up to 0.05 mm). Subscutellum dark brown, grey pruinose. Pleura dark brown. Wing. Length: 3.2–3.3 mm. LW:MWW = 2.8. Wing almost entirely covered with microtrichia. Pterostigma dark-brown and complete. LS:LTC = 1.0. LTC:LFC = 1.1. Cross-vein r-m reaches dm shortly after one-third of the cell’s length. M_1_ strongly undulating in middle. Halter length: 0.5 mm, base dark, stem and knob narrowly light brown. **Legs.** Coxae dark brown, grey pruinose. Trochanters somewhat light brown partly grey pruinose. Femora brown with pale apices, grey pruinose. Mid and hind femora bearing two rows of dark anteroventral small spines in apical half. Tibiae light brown, grey pruinose, with two rows of short setae on anterior side and three rows on posterior. Hind tibia with one or two wrinkled indentations in middle without erect anteromedial setae. Tarsi yellowish with scattered dark setae at anterior margin. Pulvilli yellow. Claws brown with black tips. **Abdomen.** Ground colour dark brown, tergite 1 silver-grey pruinose, with three long (up to 0.2 mm) and two short (up to 0.08 mm) dark lateral bristles. Tergite 2 silver-grey pruinose. Tergites 3-5 brown pruinose with scattered brown setae. Sternites white-yellow laterally with dark mid-line centrally, grey pruinose. Syntergosternite 8 enlarged, dark brown and grey pruinose. Membranous area small. **Genitalia**. Genital capsule in dorsal view: epandrium dark brown and surstyli light brown, grey pruinose. Epandrium longer than wide (MLE:MWE = 1.59) (Fig. 10A). Surstyli perpendicular to the epandrium. Right surstylus broader than left surstylus at the base, with two finger-like projections at the apex, inner finger-like projection longer than the outer one and slightly curved towards left surstylus (Fig. 10B). Left surstylus with an inner finger-like projection at the apex which is slightly broader and longer than the inner finger-like projection of right surstylus (Fig. 10B). Genital capsule in ventral view: subepandrial sclerite wide (Fig. 10C). Gonopods unequal, right gonopod slightly higher than the left one (Fig. 10C), left gonopod with a long finger-like projection towards surstyli in ventrolateral view (Fig. 10D). Genital capsule in lateral view: phallus trifid, long and circular, with strong membranous sheath; phallic guide strong, divided at base, right phallic guide with a projection in the middle which is divided by two branches, a downward branch being longer than upward one (Fig. 10E, G); ejaculatory apodeme spade-shaped (Fig. 10F).

**Female**. Body length (excluding antennae): 3.0 mm (n = 2). Eyes separated. Scape and pedicel dark brown; flagellum light brown, long tapering. Frons grey pruinose. Occiput grey pruinose. Postpronotal lobe light brown with 3-4 bristles along upper margin (up to 0.05 mm). Scutum black, brown pruinose with scattered setae at anterior supra-alar area. Wing Length: 3.2 mm. LW:MWW = 2.0. Pterostigma light-brown and complete (LS:LTC = 1.0, LTC:LFC = 1.0). Coxae and trochanters dark brown. Femora, tibiae and tarsi light brown; mid coxa with 4–5 black anterior bristles; mid tibia with 3-5 long apical bristles. Femora bearing two small ventral rows of dark peg-like spines in the apical third. Tergites 1-2 grey pruinose, tergites 3–5 posterolaterally grey pruinose, otherwise brown pruinose. **Ovipositor.** Viewed laterally: base of ovipositor light brown, piercer short (LP = 0.3 mm) and dark brown, base of piercer straight, distinctly bent towards sternite in distal third and reaching sternite 4. LP:LB = 2.0. LDP:LPP = 1.25(Fig. 11A, B).

#### Diagnosis

This species can be distinguished by the specific shape of the phallic guide, divided at the base, right phallic guide with two branched projections in the middle (Fig. 10D); strong membranous sheath (Fig. 10E, G), left gonopod with a long upward finger-like projection (Fig. 10D).

#### Etymology

The specific epithet is derived from the Latin corniculans, the diminutive form of cornuatus which means horned and references the shape of the phallic guide.

#### Distribution

Iran, Israel (Fig. 12).

#### Notes

Based on DNA barcodes, *Eudorylascorniculans* sp. n. is genetically most similar to *E.bihamatus* sp. n. (5.26% pairwise divergence).

### Eudorylas fascipes

(Zetterstedt, 1844)

218A3EB1-9B36-5D95-B033-F3B8907D6F63

Pipunculus fascipes Zetterstedt, 1844: 964

#### Diagnosis

This species can be recognised by the triangular-shaped left surstylus and broad base of right surstylus with inner finger-like projection in dorsal view; gonopods unequal, right larger than left one in ventral view; phallic guide bent with two small lobes bearing some short setae in lateral view (for illustration, see Kehlmaier 2005a: Figure 30a, h).

#### Distribution

Czech Republic, Finland, Iran (Fig. 1), Italy, Russia, Sweden (Kehlmaier 2005a, Kazerani et al. 2017, Skevington 2020).

#### Notes

No sequence data exist for this species.

### Eudorylas flavicrus

De Meyer, 1995

8D3F1617-4604-5572-B235-3ACBE7DB36D5

#### Materials

- **Type status:** Other material. **Occurrence:** catalogNumber: JSS50774; recordedBy: A. Maklakov; individualCount: 1; sex: male; lifeStage: adult; **Taxon:** scientificName: *Eudorylasflavicrus*; **Location:** country: Israel; locality: Hazeva Field Scholl; decimalLatitude: 30.716667; decimalLongitude: 35.25; **Event:** samplingProtocol: Malaise trap; eventDate: 1997-12-23; **Record Level:** institutionCode: CNC
- **Type status:** Other material. **Occurrence:** catalogNumber: JSS50800; recordedBy: A. Maklakov; individualCount: 1; sex: male; lifeStage: adult; **Taxon:** scientificName: *Eudorylasflavicrus*; **Location:** country: Israel; locality: Hazeva Field School; decimalLatitude: 30.716667; decimalLongitude: 36.25; **Event:** samplingProtocol: Malaise trap; eventDate: 1997-12-14; **Record Level:** institutionCode: TAU
- **Type status:** Other material. **Occurrence:** catalogNumber: JSS50819; recordedBy: M. E. Irwin; individualCount: 1; sex: male; lifeStage: adult; **Taxon:** scientificName: *Eudorylasflavicrus*; **Location:** country: Israel; locality: Arava Valley, En Yahav Makhteshim Res, En shohak; decimalLatitude: 30.7; decimalLongitude: 35.183333; **Event:** samplingProtocol: Malaise trap; eventDate: 1995-03-25; **Record Level:** institutionCode: TAU
- **Type status:** Other material. **Occurrence:** catalogNumber: JSS50831; recordedBy: A. Freidberg; individualCount: 1; sex: male; lifeStage: adult; **Taxon:** scientificName: *Eudorylasflavicrus*; **Location:** country: Israel; locality: Enot Zuqim; decimalLatitude: 30.483333; decimalLongitude: 35.15; **Event:** eventDate: 2002-12-23; **Record Level:** institutionCode: TAU

#### Diagnosis

This species can be recognised by asymmetrical surstyli, right surstylus quadratic-shaped at the base with a small finger-like projection apically towards left surstylus, left surstylus slightly rectangular-shape at the base with a triangular projection in dorsal view (Fig. 13A); phallic guide short, apically with downwards hook-like projection in lateral view (Fig. 13B); gonopods slightly unequal, right higher than left one in ventral view (Fig. 13B). The genitalia of this species are similar to *E.fluviatilis* (Becker, 1900). It differs by the shape of left surtylus in dorsal view and shape of right gonopod in ventral view. In *E.flavicrus*, the left surstylus has a rather rectangular base followed by a triangular projection in dorsal view (Fig. 13a) and the right gonopod has a distinct finger-like projection (Fig. 13B).

#### Distribution

Israel (Fig. 6).

#### Notes

No sequence data exist for this species.

### Eudorylas fuscipes

(Zetterstedt, 1844)

1B80075B-BCC2-5C63-8B0F-9EEE53BC9828

Pipunculus fuscipes Zetterstedt, 1844:953

#### Diagnosis

This species can be recognised by the very large membranous area; rather symmetrical surstyli in dorsal view, left surstylus with squared base, right one with base longer than wide, both surstyli with a rather inner finger-like process in dorsal view; gonopods small, left is higher than right one; phallic guide broad and straight with inner lateral margin distinctly convex, outer lateral margin slightly convex to straight and pointed apex in ventral view (for illustration, see Kehlmaier 2005a: Figure 37a, k).

#### Distribution

**Figure 6.**
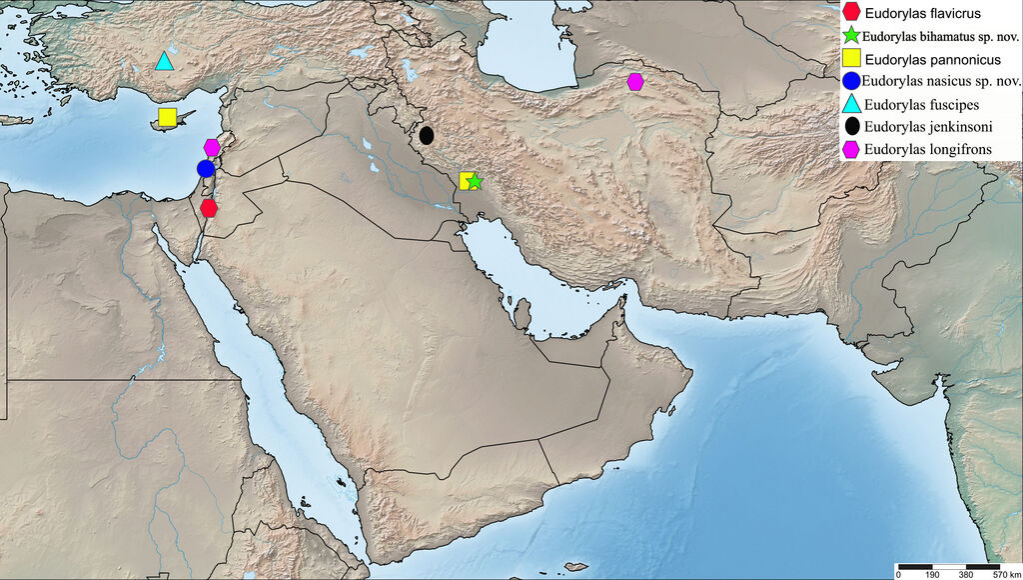
*Eudorylas* species distribution in the Middle East.

**Figure 7.**
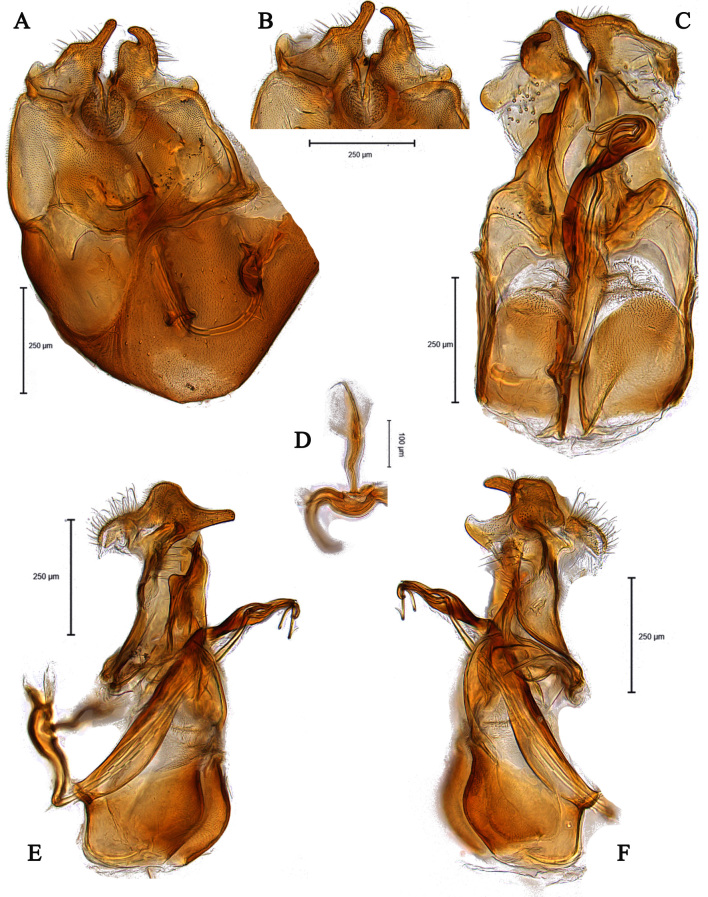
Male genitalia of *Eudorylasbipertitus* (JSS50815) (A) in dorsal view; (B) surstyli in dorsal view; (C) in ventral view; (D) ejaculatory apodeme; (E, F) in lateral view.

**Figure 8.**
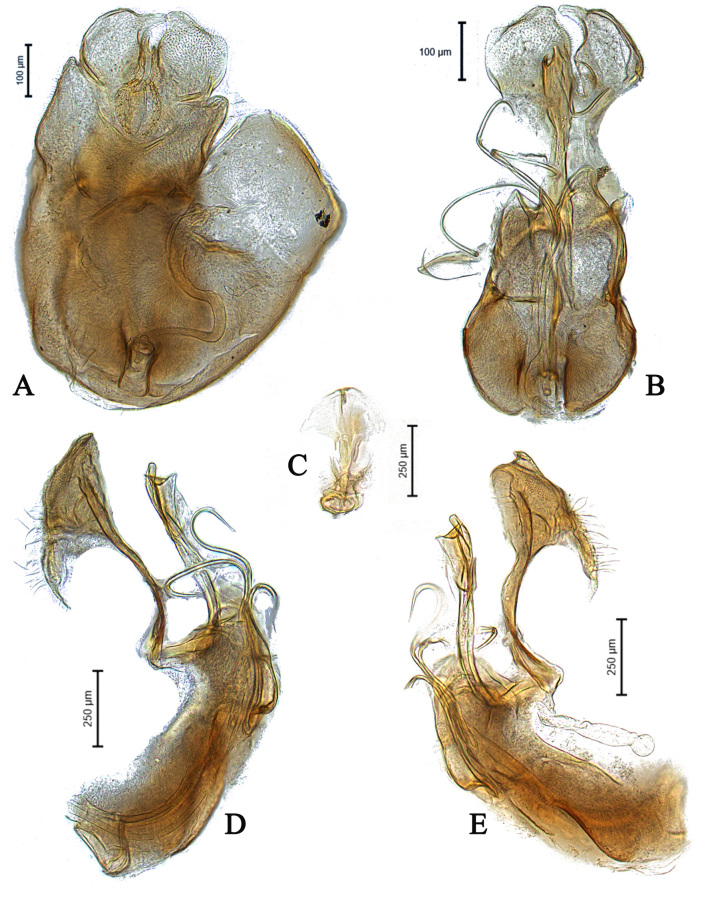
Male genitalia of *Eudorylasblascoi* (JSS52179) (A) in dorsal view; (B) in ventral view; (C) ejaculatory apodeme; (D, E) in lateral view.

**Figure 9.**
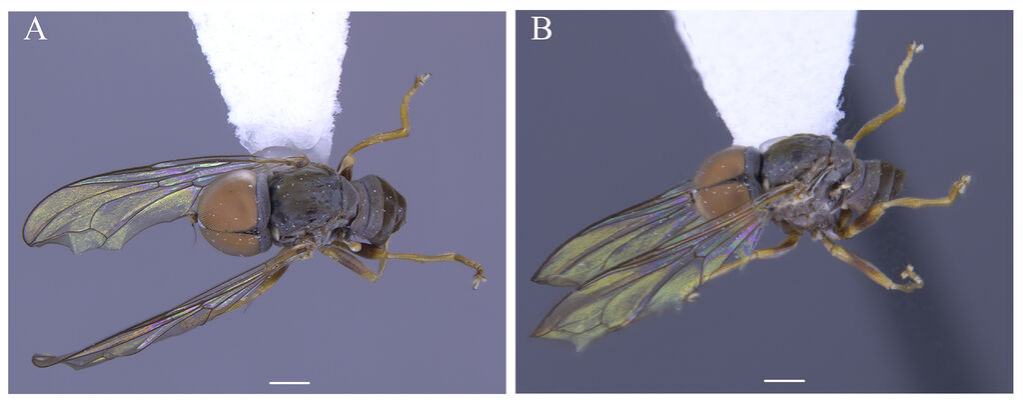
Male of *Eudorylascorniculans* Motamedinia & Skevington sp. n. (JSS52187; most of abdomen removed for terminalia dissection) (A) habitus in dorsal view; (B) habitus in lateral view. Scale bar = 1 mm.

**Figure 10.**
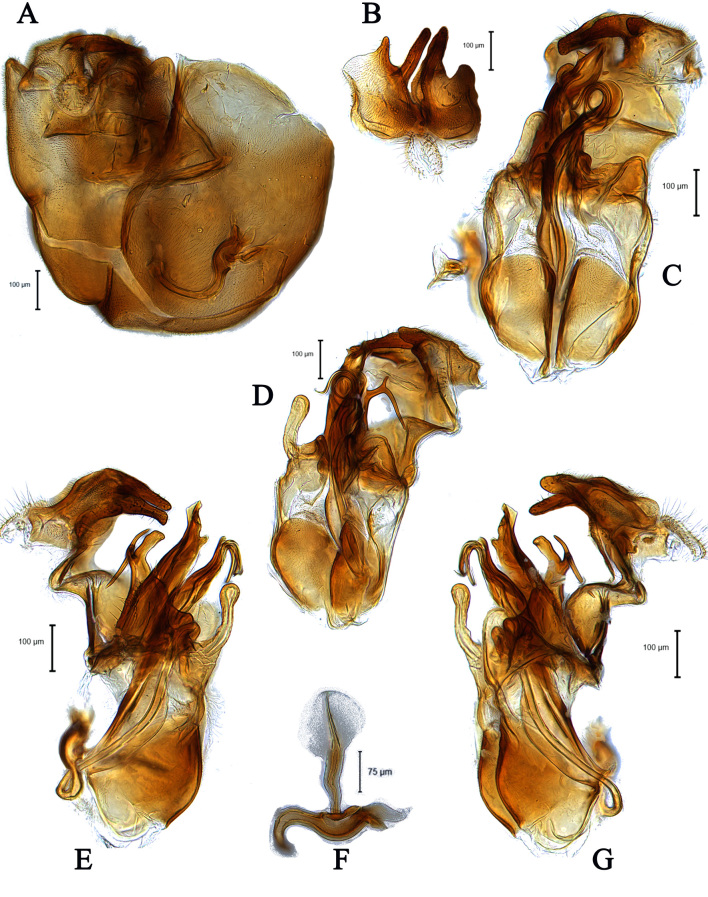
Male genitalia of *Eudorylascorniculans* Motamedinia & Skevington sp. n.(JSS52187) (A) in dorsal view; (B) surstyli in dorsal view; (C) in ventral view; (D) in lateral-ventral view; (E,G) in lateral view; (F) ejaculatory apodeme.

**Figure 11.**
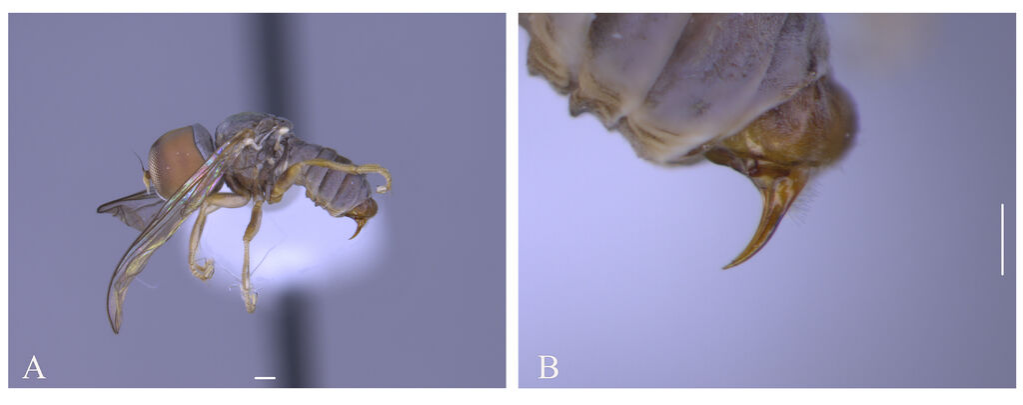
Female of *Eudorylascorniculans* Motamedinia & Skevington sp. n. (JSS52206) (A) habitus in lateral view; (B) ovipositor in lateral view. Scale bar = 0.25 mm.

Austria, Belgium, Bulgaria, Croatia, Czech Republic, Denmark, England, Finland, Germany, Hungary, Ireland, Italy, Latvia, Macedonia, Netherlands, North Korea, Poland, Russia, Slovakia, Slovenia, Spain, Sweden, Switzerland, Turkey (Fig. 6) (Kehlmaier 2005a, Skevington 2020).

#### Notes

*Eudorylasfuscipes* is genetically closest to an undescribed Chinese *Eudorylas* species (*E.* sp. China13) differing by 3.77-4.01% (pairwise divergence). It is 3.51%- 4.9% different from *E.zonellus* Collin, 1956 and 5.90% from *E.montium* (Becker, 1898).

### Eudorylas fluviatilis

(Becker, 1900)

8F8B0892-223A-5860-8D0C-008179AA0A54

Pipunculus fluviatilis Becker, 1900: 224

#### Materials

- **Type status:** Other material. **Occurrence:** catalogNumber: JSS50763; recordedBy: A. Freidberg; individualCount: 1; sex: male; lifeStage: adult; associatedSequences: GB: MN549649; **Taxon:** scientificName: *Eudorylasfluviatilis*; **Location:** country: Israel; locality: Tel Qeshet; decimalLatitude: 31.533333; decimalLongitude: 34.766667; **Event:** eventDate: 2004-10-12; **Record Level:** institutionCode: TAU
- **Type status:** Other material. **Occurrence:** catalogNumber: JSS50768; recordedBy: A. Freidberg; individualCount: 1; sex: male; lifeStage: adult; **Taxon:** scientificName: *Eudorylasfluviatilis*; **Location:** country: Israel; locality: Tel Qeshet; decimalLatitude: 31.533333; decimalLongitude: 34.766667; **Event:** eventDate: 2004-10-12; **Record Level:** institutionCode: TAU
- **Type status:** Other material. **Occurrence:** catalogNumber: JSS50775; recordedBy: Y. Nussbaum; individualCount: 1; sex: male; lifeStage: adult; **Taxon:** scientificName: *Eudorylasfluviatilis*; **Location:** country: Israel; locality: Rehovot; decimalLatitude: 31.883333; decimalLongitude: 34.8; **Event:** eventDate: 1991-01-02; **Record Level:** institutionCode: TAU
- **Type status:** Other material. **Occurrence:** catalogNumber: JSS50779; recordedBy: A. Freidberg; individualCount: 1; sex: male; lifeStage: adult; **Taxon:** scientificName: *Eudorylasfluviatilis*; **Location:** country: Israel; locality: Herzliyya; decimalLatitude: 31.15; decimalLongitude: 34.85; **Event:** eventDate: 1995-12-02; **Record Level:** institutionCode: TAU
- **Type status:** Other material. **Occurrence:** catalogNumber: JSS50794; recordedBy: A. Freidberg; individualCount: 1; sex: male; lifeStage: adult; **Taxon:** scientificName: *Eudorylasfluviatilis*; **Location:** country: Israel; locality: Haifa; decimalLatitude: 32.791694; decimalLongitude: 34.988806; **Event:** eventDate: 1994-03-27; **Record Level:** institutionCode: TAU
- **Type status:** Other material. **Occurrence:** catalogNumber: JSS50795; recordedBy: A. Freidberg; individualCount: 1; sex: male; lifeStage: adult; **Taxon:** scientificName: *Eudorylasfluviatilis*; **Location:** country: Israel; locality: Tel Qeshet; decimalLatitude: 31.533333; decimalLongitude: 34.766667; **Event:** eventDate: 2001-10-13; **Record Level:** institutionCode: TAU
- **Type status:** Other material. **Occurrence:** catalogNumber: JSS50806; recordedBy: A. Freidberg; individualCount: 1; sex: male; lifeStage: adult; **Taxon:** scientificName: *Eudorylasfluviatilis*; **Location:** country: Israel; locality: Nahal 'lyyon Ha Tanur Waterfall; decimalLatitude: 33.266667; decimalLongitude: 35.566667; **Event:** eventDate: 2011-03-15; **Record Level:** institutionCode: TAU
- **Type status:** Other material. **Occurrence:** catalogNumber: JSS50820; recordedBy: A. Freidberg; individualCount: 1; sex: male; lifeStage: adult; **Taxon:** scientificName: *Eudorylasfluviatilis*; **Location:** country: Israel; locality: Tel Qeshet; decimalLatitude: 31.533333; decimalLongitude: 34.766667; **Event:** eventDate: 2001-10-01; **Record Level:** institutionCode: TAU
- **Type status:** Other material. **Occurrence:** catalogNumber: JSS50823; recordedBy: A. Freidberg & L. Friedman; individualCount: 1; sex: male; lifeStage: adult; **Taxon:** scientificName: *Eudorylasfluviatilis*; **Location:** country: Israel; locality: Herzliyya; decimalLatitude: 31.15; decimalLongitude: 34.85; **Event:** eventDate: 2000-12-18; **Record Level:** institutionCode: TAU
- **Type status:** Other material. **Occurrence:** catalogNumber: JSS51830; recordedBy: F. Hamzavi; individualCount: 1; sex: female; lifeStage: adult; associatedSequences: GB: MN549650; **Taxon:** scientificName: *Eudorylasfluviatilis*; **Location:** country: Iran; stateProvince: Sistan & Baluchestan; locality: Saravan; decimalLatitude: 27.416667; decimalLongitude: 62.283333; **Event:** samplingProtocol: pan trap; eventDate: 2016-11-08; **Record Level:** institutionCode: CNC
- **Type status:** Other material. **Occurrence:** catalogNumber: JSS52151; recordedBy: M. Zardouei; individualCount: 1; sex: female; lifeStage: adult; associatedSequences: GB: MN549666; **Taxon:** scientificName: *Eudorylasfluviatilis*; **Location:** country: Iran; stateProvince: Kermanshah; locality: Sarpolezahab; decimalLatitude: 34.466667; decimalLongitude: 45.816667; **Event:** samplingProtocol: Malaise trap; eventDate: 2016-09-07; **Record Level:** institutionCode: CNC
- **Type status:** Other material. **Occurrence:** catalogNumber: JSS52168; recordedBy: M. Ghaforimoghadam; individualCount: 1; sex: female; lifeStage: adult; associatedSequences: GB: MN549664; **Taxon:** scientificName: *Eudorylasfluviatilis*; **Location:** country: Iran; stateProvince: Sistan & Baluchestan; locality: Iranshahr; decimalLatitude: 27.4; decimalLongitude: 60.833333; **Event:** samplingProtocol: Malaise trap; eventDate: 2016-05-02/12; **Record Level:** institutionCode: CNC
- **Type status:** Other material. **Occurrence:** catalogNumber: JSS52195; recordedBy: M. Ghaforimoghadam; individualCount: 1; sex: male; lifeStage: adult; associatedSequences: GB: MN549661; **Taxon:** scientificName: *Eudorylasfluviatilis*; **Location:** country: Iran; stateProvince: Sistan & Baluchestan; locality: Rask; decimalLatitude: 26.266667; decimalLongitude: 61.416667; **Event:** samplingProtocol: Malaise trap; eventDate: 2016-06-10/07-14; **Record Level:** institutionCode: CNC
- **Type status:** Other material. **Occurrence:** catalogNumber: JSS52308; recordedBy: O. Ozden; individualCount: 1; sex: male; lifeStage: adult; **Taxon:** scientificName: *Eudorylasfluviatilis*; **Location:** country: Cyprus; locality: Kyrenia; decimalLatitude: 35.3477; decimalLongitude: 33.1504; **Event:** samplingProtocol: Malaise trap; eventDate: 2017-07-09/16; **Record Level:** institutionCode: CNC
- **Type status:** Other material. **Occurrence:** catalogNumber: JSS52309; recordedBy: O. Ozden; individualCount: 1; sex: male; lifeStage: adult; **Taxon:** scientificName: *Eudorylasfluviatilis*; **Location:** country: Cyprus; locality: Kyrenia; decimalLatitude: 35.3477; decimalLongitude: 33.1504; **Event:** samplingProtocol: Malaise trap; eventDate: 2017-09-24/10-01; **Record Level:** institutionCode: CNC
- **Type status:** Other material. **Occurrence:** catalogNumber: JSS52310; recordedBy: O. Ozden; individualCount: 1; sex: male; lifeStage: adult; **Taxon:** scientificName: *Eudorylasfluviatilis*; **Location:** country: Cyprus; locality: Kyrenia; decimalLatitude: 35.3477; decimalLongitude: 33.1504; **Event:** samplingProtocol: Malaise trap; eventDate: 2017-10-22/29; **Record Level:** institutionCode: CNC
- **Type status:** Other material. **Occurrence:** catalogNumber: JSS52311; recordedBy: O. Ozden; individualCount: 1; sex: male; lifeStage: adult; **Taxon:** scientificName: *Eudorylasfluviatilis*; **Location:** country: Cyprus; locality: Kyrenia; decimalLatitude: 35.3477; decimalLongitude: 33.1504; **Event:** samplingProtocol: Malaise trap; eventDate: 2017-10-22/29; **Record Level:** institutionCode: CNC
- **Type status:** Other material. **Occurrence:** catalogNumber: JSS50770; individualCount: 1; sex: male; lifeStage: adult; **Taxon:** scientificName: *Eudorylasfluviatilis*; **Location:** country: Israel; locality: Park haYarden; decimalLatitude: 32.9; decimalLongitude: 35.616667; **Record Level:** institutionCode: TAU
- **Type status:** Other material. **Occurrence:** catalogNumber: JSS50803; recordedBy: L. Friedman; individualCount: 1; sex: male; lifeStage: adult; **Taxon:** scientificName: *Eudorylasfluviatilis*; **Location:** country: Israel; locality: Nizzanim, Nature Reserve Nahal Evatah; decimalLatitude: 31.75; decimalLongitude: 34.633333; **Event:** eventDate: 2008-07-28; **Record Level:** institutionCode: TAU
- **Type status:** Other material. **Occurrence:** catalogNumber: JSS51914; recordedBy: H. Davari; individualCount: 1; sex: female; lifeStage: adult; associatedSequences: GB: MN549660; **Taxon:** scientificName: *Eudorylasfluviatilis*; **Location:** country: Iran; stateProvince: Sistan & Baluchestan; locality: Iranshahr, Daman; decimalLatitude: 27.4; decimalLongitude: 60.833333; **Event:** samplingProtocol: Malaise trap; eventDate: 2016-04-02/12; **Record Level:** institutionCode: CNC
- **Type status:** Other material. **Occurrence:** catalogNumber: JSS51915; recordedBy: H. Davari; individualCount: 1; sex: female; lifeStage: adult; **Taxon:** scientificName: *Eudorylasfluviatilis*; **Location:** country: Iran; stateProvince: Sistan & Baluchestan; locality: Iranshahr, Daman; decimalLatitude: 27.4; decimalLongitude: 60.833333; **Event:** samplingProtocol: Malaise trap; eventDate: 2016-04-02/12; **Record Level:** institutionCode: CNC
- **Type status:** Other material. **Occurrence:** catalogNumber: JSS52160; recordedBy: F. Hamzavi; individualCount: 1; sex: male; lifeStage: adult; associatedSequences: GB: MN549652; **Taxon:** scientificName: *Eudorylasfluviatilis*; **Location:** country: Iran; stateProvince: Sistan & Baluchestan; locality: Saravan; decimalLatitude: 27.416667; decimalLongitude: 62.283333; **Event:** samplingProtocol: Malaise trap; eventDate: 2016-11-08; **Record Level:** institutionCode: CNC

#### Diagnosis

The male of this species can be recognised by the small membranous area; unequal gonopods in ventral view (Fig. 14A); right surstylus apically with two short projection in lateral view (Fig. 14E); left surstylus apically with long projection in lateral view (Fig. 14D); phallic guide with hook pointing downwards in ventral view (Fig. 14B).

#### Distribution

Cyprus, Egypt, France, Greece, Iran, Israel, Russia, Spain, Turkey (Kehlmaier 2005a, Motamedinia et al. 2017, Kehlmaier et al. 2019, Skevington 2020) (Fig. 1).

#### Notes

*Eudorylasfluviatilis* is in the *E.mutillatus* species complex. It is genetically close to specimens from Indonesia, Bangladesh, China, Vietnam, Australia, Israel, Egypt, Yemen and Pakistan, differring by 0.77-4.02%. The genitalia are very similar between *E.fluviatilis* and *E.mutillatus* (Loew, 1858), raising the possibility that the two taxa are conspecific. *Eudorylasmutillatus* was illustrated and re-described by Skevington (2002a) and Földvári (2013), but this species must be re-assessed across its entire range. A second genetic marker should be used in conjunction with COI to test the species concept (likely ITS2) and to examine closely-related and potentially-synonymous species like *E.fluviatilis*.

### Eudorylas gemellus

Kehlmaier, 2005

FE3178F8-15C7-5921-A8AB-55C73A9F2BC5

#### Materials

- **Type status:** Other material. **Occurrence:** catalogNumber: JSS50796; recordedBy: A.Freidberg; sex: male; lifeStage: adult; associatedSequences: GB: MN549659; **Taxon:** scientificName: *Eudorylasgemellus*; **Location:** country: Israel; locality: Karmel; decimalLatitude: 32.733333; decimalLongitude: 35.033333; **Record Level:** institutionCode: TAU

#### Diagnosis

This species can be recognised by a long phallic guide in ventral view (Fig. 15B); epandrium longer than wide (Fig. 15A); surstyli with small finger-like projection apically in dorsal view (Fig. 15A); gonopods asymmetrical and small with a small-sized hump-like projections on each side (Fig. 15B); subepandrial sclerite wide with scattered setae in ventral view (Fig. 15B).

#### Distribution

**Figure 12.**
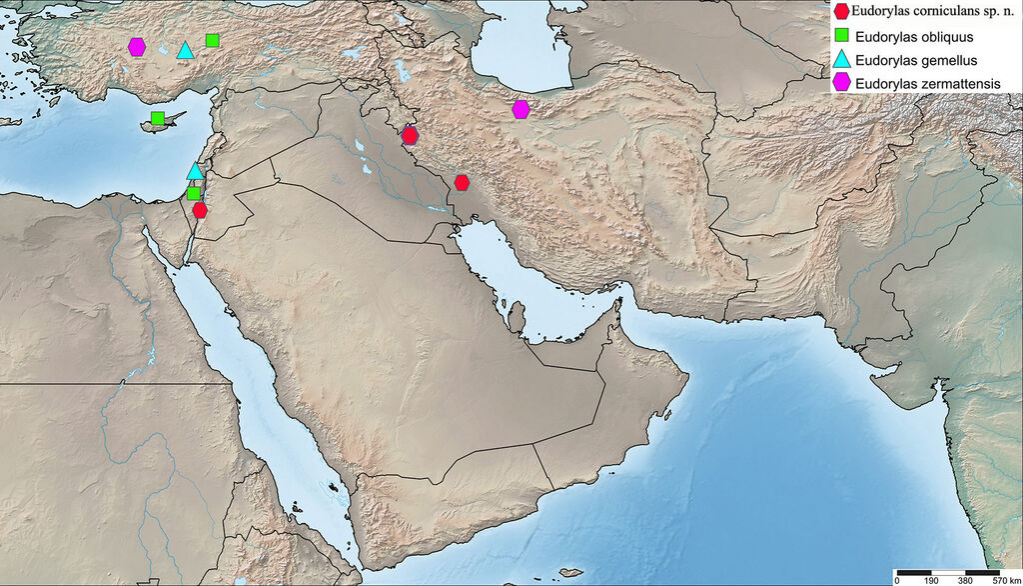
*Eudorylas* species distribution in the Middle East.

**Figure 13.**
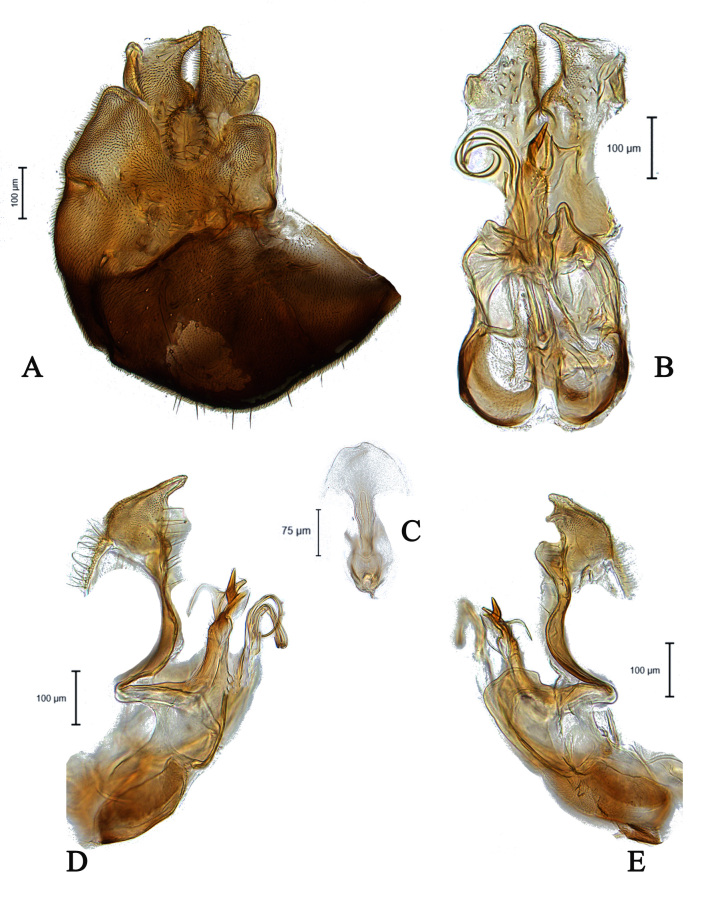
Male genitalia of *Eudorylasflavicrus* (JSS50774) (A) in dorsal view; (B) in ventral view; (C) ejaculatory apodeme; (D, E) in lateral view.

**Figure 14.**
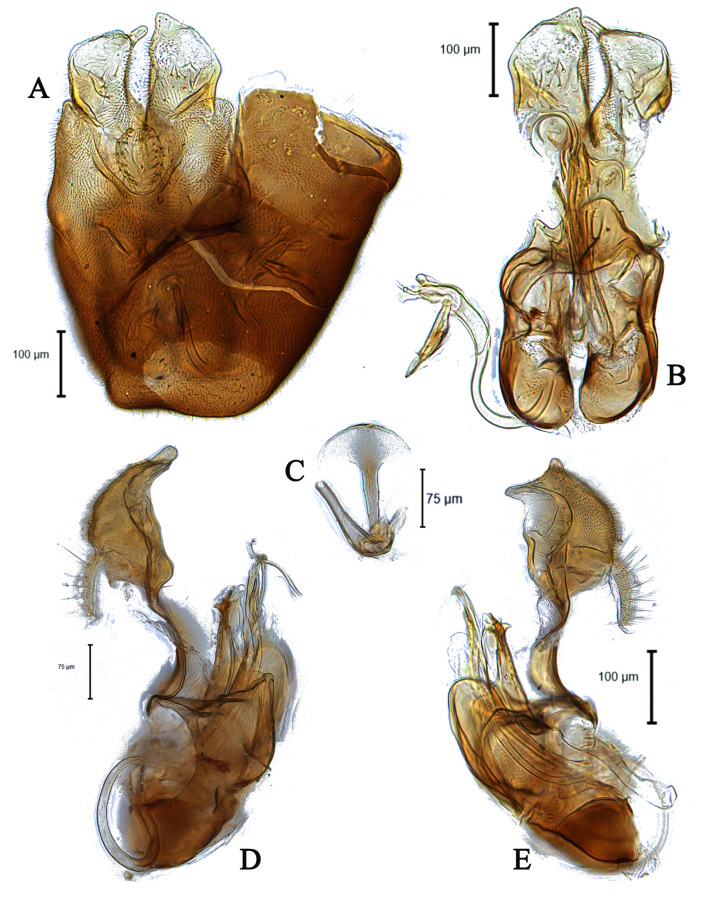
Male genitalia of *Eudorylasfluviatilis* (JSS52195) (A) in dorsal view; (B) in ventral view; (C) ejaculatory apodeme; (D, E) in lateral view.

**Figure 15.**
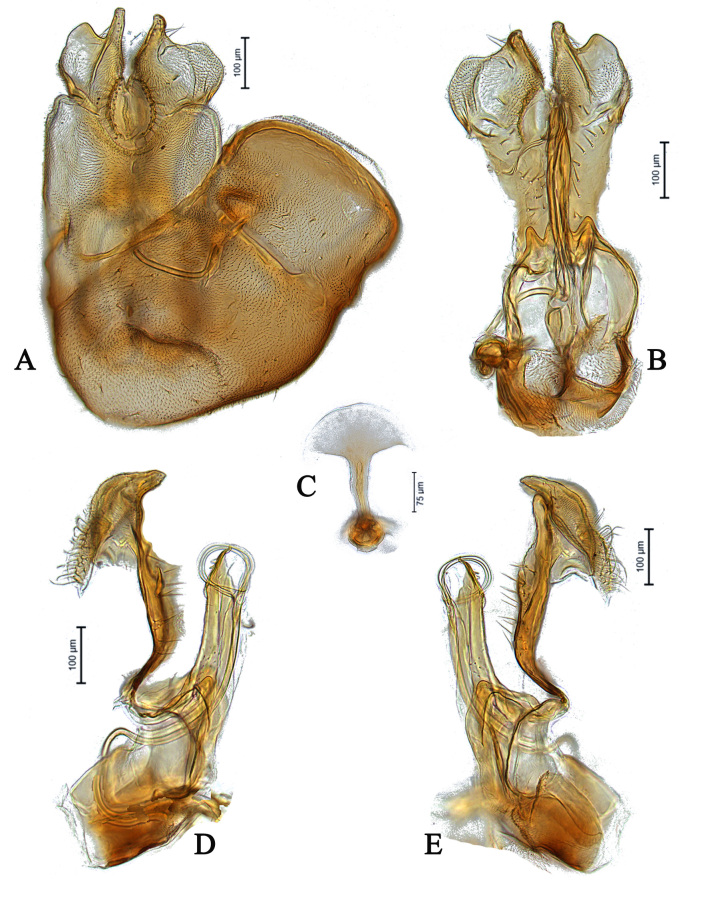
Male genitalia of *Eudorylasgemellus* (JSS50796) (A) in dorsal view; (B) in ventral view; (C) ejaculatory apodeme; (D, E) in lateral view.

Croatia, Czech Republic, France, Israel, Italy, Switzerland, Turkey (Kehlmaier 2005a, Skevington 2020) (Fig. 12).

#### Notes

*Eudorylasgemellus* is genetically similar to *E.arcanus* Coe, 1966 (6.71-7.15% pairwise divergence) and the *E.obscurus* complex (including *E.auctus* and *E.longifrons* differing by 4.55% and 4.78%, respectively).

### Eudorylas jenkinsoni

Coe, 1966

77908663-E70A-53AE-9AC6-71484CFF3F11

#### Diagnosis

This species can be recognised by the size of the right surstylus in dorsal view, wider than long with inner finger-like projection; left surstylus triangular-shaped in dorsal view and dorsal margin of left surstylus humped in lateral view; gonopods small and equal in height; phallic guide short and straight with two triangular projection dorsomedially in lateral view (for illustration, see Kehlmaier, 2005: Fig. 31a, n).

#### Distribution

Belgium, Bulgaria, Czech Republic, Denmark, England, Finland, France, Germany, Hungary, Iran (Fig. 6), Italy, Japan, Latvia, Netherlands, Norway, Poland, Portugal, Slovakia, Sweden, Switzerland (Kehlmaier 2005a, Motamedinia et al. 2017, Skevington 2020).

#### Notes

DNA barcodes of *Eudorylasjenkinsoni* overlap with those of *E.obliquus* (0.62-1.63% pairwise divergence). The genitalia of these species differ by the size of the right surstylus in dorsal view, wider than long in *E.jenkinsoni*, so this is likely another case of recently-diverged species or ancestral hybridisation. There is always a possibility that it is a single species with polymorphic genitalia, so future genetic work is warranted.

### Eudorylas longifrons

Coe, 1966

1B43BABB-A6C8-5A0D-94C7-A6525186090B

#### Diagnosis

This species can be recognised by asymmetrical surstyli with short inner finger-like projection, right longer than left one in dorsal view; gonopods small, right slightly higher than left one in ventral view; phallic guide straight and broad in lateral view (for illustration, see Kehlmaier 2005a: Fig. 50a, l).

#### Distribution

Belgium, Croatia, Czech Republic, Denmark, France, Germany, England, Hungary, Iran, Israel, Italy, Latvia, Macedonia, Romania, Slovakia, Switzerland (Kehlmaier 2005a, Skevington 2020) (Fig. 6).

#### Notes

DNA barcodes of *Eudorylaslongifrons* overlap with *E.auctus* and *E.obscurus*. See the notes under *E.auctus* for more details.

### Eudorylas nasicus

Motamedinia & Skevington, 2020 sp. n.

7B5D9EDD-C989-5BAD-B19E-E131A2419EBF

3BCDC8FF-F797-44F2-9D1A-7D925F876CA5

#### Materials

- **Type status:** Holotype. **Occurrence:** catalogNumber: JSS50793; recordedBy: A. Freidberg; individualCount: 1; sex: Male; associatedSequences: GB: MN549667; **Location:** country: Israel; locality: Zomet Ha'Amaqim (Jalame); decimalLatitude: 32.716; decimalLongitude: 35.10; **Event:** eventDate: 1993-05-18/22; **Record Level:** institutionCode: TAU

#### Description

**Figure 16.**
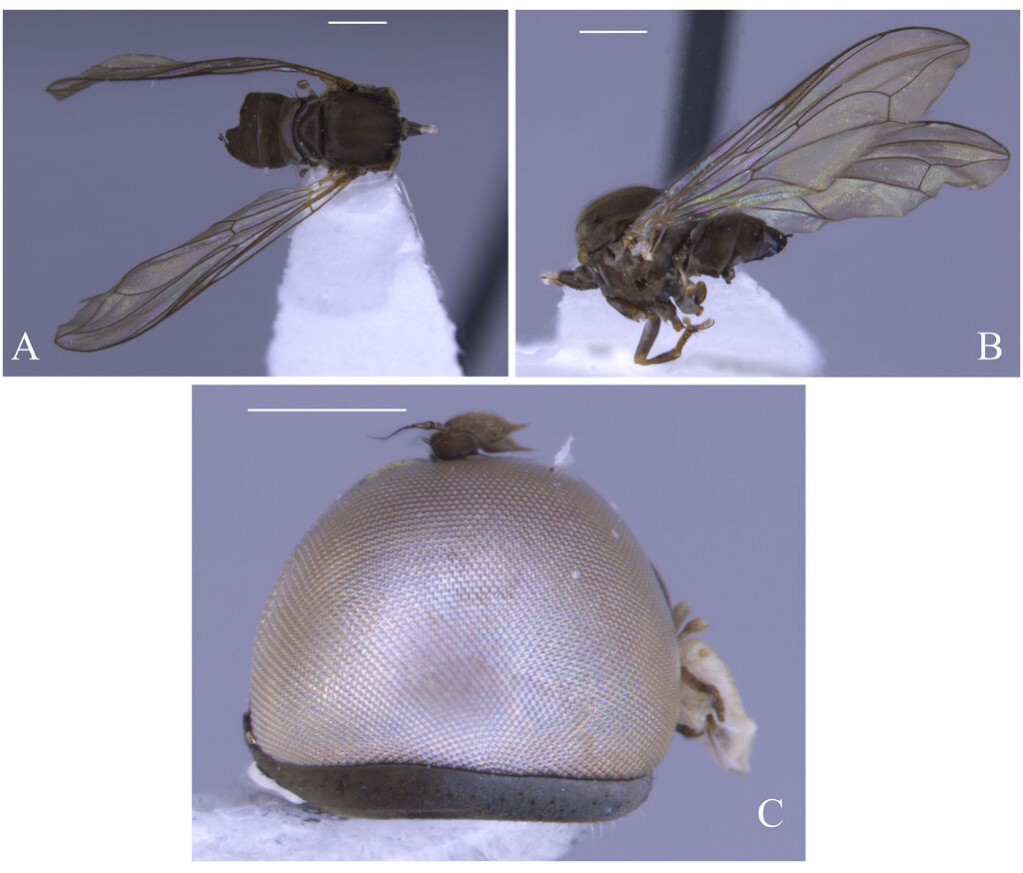
Male of *Eudorylasnasicus* Motamedinia & Skevington sp. n. (JSS50793) (A) habitus in dorsal view, scale bar = 1 mm; (B) habitus in lateral view, scale bar = 1 mm; (C) compound eyes in lateral view, scale bar = 500 µm.

**Figure 17.**
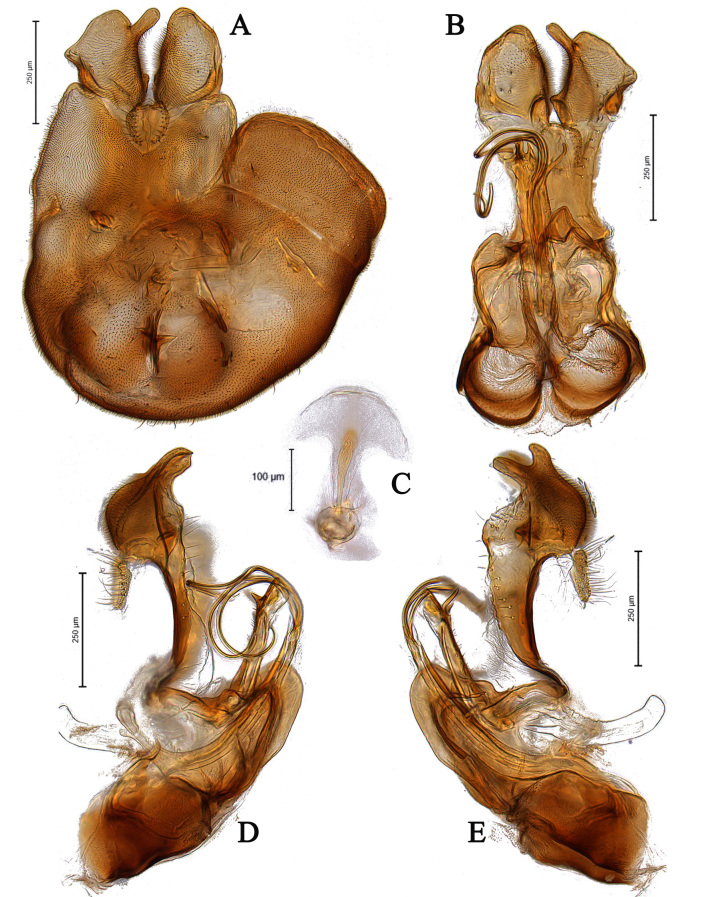
Male genitalia of *Eudorylasnasicus* sp. n. (JSS50793) (A) in dorsal view; (B) in ventral view; (C) ejaculatory apodeme; (D, E) in lateral view.

**Male** (Fig. 16A-C). Body length (excluding antennae): 3.9 mm. **Head.** Scape dark with 1-2 dark upper bristles, pedicel brown with two long and two short upper bristles and two long lower bristles, flagellum tapering and brown pruinose (LF:WF = 3.0); arista with thickened base. Eyes meeting for a distance of 15-17 facets. Frons dark brown pruinose with a weak median shining tubercle. Vertex black, bearing an elevated ocellar triangle. Occiput dark with scattered dark bristles. **Thorax.** Postpronotal lobe light brown, grey pruinose and with 2-4 postpronotal light brown bristles along upper margin (up to 0.05 mm). Prescutum and scutum black, predominantly brown pruinose, with two uniseriate dorsocentral rows of dark bristles and longer supra-alar bristles. Scutellum black, brown pruinose with 14 thin short setae along posterior margin (up to 0.05 mm). Subscutellum and pleura dark brown, grey pruinose. Wing. Length: 4.1 mm. LW:MWW = 4.0. Wing almost entirely covered with microtrichia. Pterostigma dark-brown and complete. LS:LTC = 1.0. LTC:LFC = 1.1. Cross-vein r-m reaches dm shortly after one-third of the cell’s length. M_1_ moderately undulating in middle. Halter length: 0.5 mm, base and knob dark brown, stem narrowly light brown. **Legs.** Coxae dark brown, grey pruinose. Fore and hind coxae with four to five short brown setae and mid coxa with two long dark setae and three brown setae on inner apical corner. Trochanters somewhat light brown partly grey pruinose. Fore femur dark brown with pale apices, grey pruinose bearing two rows of dark anteroventral small spines in apical half. Tibiae dark brown, grey pruinose, with two rows of short setae on anterior side and three rows on posterior. Tarsi light brown with scattered dark setae at anterior margin. Pulvilli yellow. Claws light brown with black tips. **Abdomen.** Ground colour dark brown, tergite 1 brown pruinose, with three to four long dark (up to 0.16 mm) and five to six short (up to 0.08 mm) brown lateral bristles. Tergites 2-5 brown pruinose with scattered brown setae. Sternites brown with some scattered dark setae. Membranous area ovate. **Genitalia**. Genital capsule in dorsal view: epandrium brown, grey pruinose. Epandrium longer than wide (MLE:MWE = 1.2) (Fig. 17A). Both surstyli rather rectangular-shaped at base. Left surstylus triangular-shaped in apical one third. Right surstylus broader than left one, with a small projection at outer margin in middle and with a longer finger-like projection at inner margin shortly before its apex, pointing towards left surstylus (Fig. 17A). Genital capsule in ventral view: subepandrial sclerite wide (Fig. 17B). Gonopods unequal, right gonopod higher than the left one. Phallus trifid; phallic guide short, broad and straight, with a long lateral projection towards right gonopod horizontally and bent upwards at its apex (Fig. 17B). Genital capsule in lateral view: phallic guide straight with a small dorsolateral projection before its apex (Fig. 17D). Left surstylus rounded with a finger-like projection apically and with a ventral margin distinctly concave in apical one third (Fig. 17D). Right surstylus broadened at base with two finger-like projection apically, the longer one is situated ventrally before the apex and the shorter one arising from the apex (Fig. 17E). Ejaculatory apodeme spade-shaped (Fig. 17C).

#### Diagnosis

This species can be recognised by asymmetrical surstyli in dorsal view, both surstyli rather rectangular-shaped at base, left surstylus triangular-shaped in apical one third, right surstylus with an inner finger-like projection shortly before its apex, pointing towards left surstylus in dorsal view and with small projection at outer margin in middle (Fig. 17A); gonopods unequal, right slightly higher than left one in ventral view (Fig. 17B); phallus trifid; phallic guide short and straight, with a long lateral projection towards right gonopod horizontally and bent upwards at its apex in ventral view (Fig. 17B). Phallic guide with a small but distinct dorsal projection shortly before its apex in lateral view (Fig. 17D, E).

#### Etymology

The specific epithet is derived from the Latin nasicus (= nose), referring to the long projection of the right surstylus in dorsal view.

#### Distribution

Israel (Fig. 6).

#### Taxon discussion

**Figure 18.**
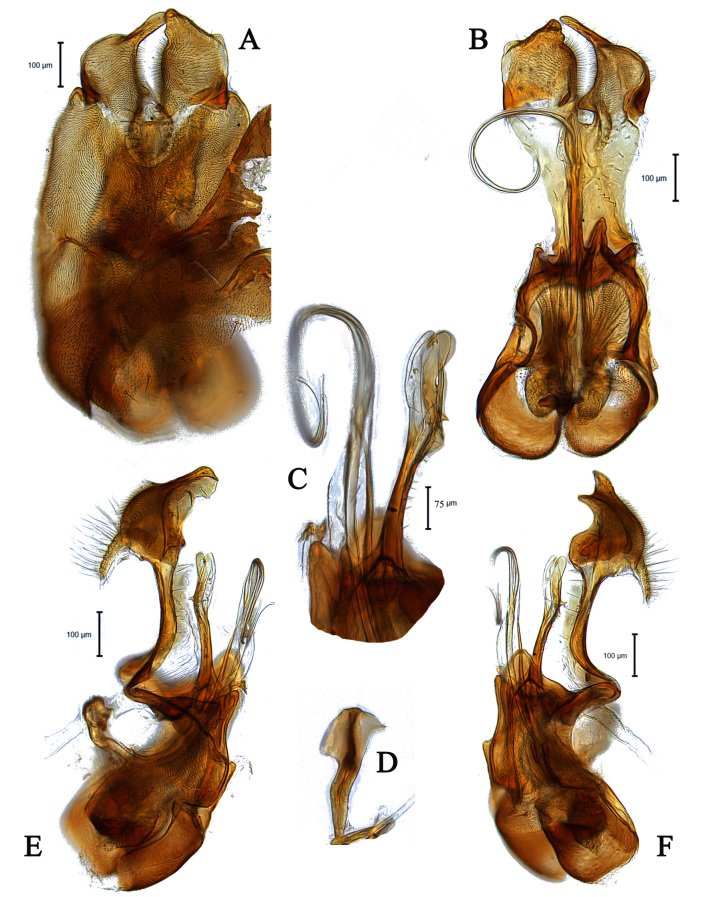
Male genitalia of *Eudorylaspannonicus* (JSS52207) (A) in dorsal view; (B) in ventral view; (C) phallus & phallic guide in ventrolateral view; (D) ejaculatory apodeme; (E,F) in lateral view.

Male *E.nasicus* sp. n. can be identified by the shape of surstyli and phallic guide, which place it in close relation to *E.unicolor, E.wahisi* and *E.pannonicus.* The right surstylus of all four of these species shows an inner finger-like projection in dorsal view and the phallic guide has a distinct projection in ventral view. Compared to *E.unicolor*, the right surstylus of *E.nasicus* sp. n. has a distinct small projection at the outer margin in the middle in dorsal view (Fig. 17A), whereas in *E.unicolor*, it does not have a distinct small projection at the outer margin in the middle (see Kehlmaier 2005a: Fig. 67j). Meanwhile, in *E.nasicus* sp. n., the left surstylus in lateral view is rounded (circle-shaped), with a finger-like projection apically and with a ventral margin distinctly concave in the apical one third (Fig. 17D), whereas in *E.unicolor*, the left surstylus is not rounded and has the ventral margin distinctly concave from the base to the apex. In *E.wahisi*, the finger-like projection of right surstylus is wider and the lateral projection of the phallic guide is long (see Kehlmaier 2005a: Fig. 68a, j). In *E.pannonicus*, the finger-like projection of right surstylus is longer and the right gonopod has two distinct projections (Fig. 18B).

#### Notes

DNA barcodes of *Eudorylasnasicus* sp. n. and *E.pannonicus* are similar. See the notes under *E.pannonicus*.

### Eudorylas obliquus

Coe, 1966

809F6326-DD41-528A-A7A8-9A05B5F949B7

Eudorylas obliquus Coe, 1966: 70

#### Materials

- **Type status:** Other material. **Occurrence:** catalogNumber: JSS50760; recordedBy: A. Freidberg; individualCount: 1; sex: male; lifeStage: adult; **Taxon:** scientificName: *Eudorylasobliquus*; **Location:** country: Israel; locality: Zikhron Ya'aqov; decimalLatitude: 32.566667; decimalLongitude: 34.95; **Event:** eventDate: 1998-04-01; **Record Level:** institutionCode: TAU
- **Type status:** Other material. **Occurrence:** catalogNumber: JSS50762; recordedBy: A. Freidberg; individualCount: 1; sex: male; lifeStage: adult; **Taxon:** scientificName: *Eudorylasobliquus*; **Location:** country: Israel; locality: Berekhat Ya'ar; decimalLatitude: 32.416667; decimalLongitude: 34.883333; **Event:** eventDate: 2004-04-26; **Record Level:** institutionCode: TAU
- **Type status:** Other material. **Occurrence:** catalogNumber: JSS50764; recordedBy: A. Freidberg; individualCount: 1; sex: male; lifeStage: adult; associatedSequences: GB: MN549651; **Taxon:** scientificName: *Eudorylasobliquus*; **Location:** country: Israel; locality: Herzliyya; decimalLatitude: 31.15; decimalLongitude: 34.85; **Event:** eventDate: 2005-04-08; **Record Level:** institutionCode: TAU
- **Type status:** Other material. **Occurrence:** catalogNumber: JSS50765; recordedBy: A. Freidberg; individualCount: 1; sex: male; lifeStage: adult; **Taxon:** scientificName: *Eudorylasobliquus*; **Location:** country: Israel; locality: Herzliyya; decimalLatitude: 31.15; decimalLongitude: 34.85; **Event:** eventDate: 2005-04-08; **Record Level:** institutionCode: TAU
- **Type status:** Other material. **Occurrence:** catalogNumber: JSS50766; recordedBy: A. Freidberg; individualCount: 1; sex: male; lifeStage: adult; **Taxon:** scientificName: *Eudorylasobliquus*; **Location:** country: Israel; locality: Herzliyya; decimalLatitude: 31.15; decimalLongitude: 34.85; **Event:** eventDate: 2005-04-08; **Record Level:** institutionCode: TAU
- **Type status:** Other material. **Occurrence:** catalogNumber: JSS50767; recordedBy: A. Freidberg; individualCount: 1; sex: male; lifeStage: adult; **Taxon:** scientificName: *Eudorylasobliquus*; **Location:** country: Israel; locality: Bet Guvrin; decimalLatitude: 31.6; decimalLongitude: 34.883333; **Event:** eventDate: 2004-03-10; **Record Level:** institutionCode: TAU
- **Type status:** Other material. **Occurrence:** catalogNumber: JSS50769; recordedBy: A. Freidberg; individualCount: 1; sex: male; lifeStage: adult; **Taxon:** scientificName: *Eudorylasobliquus*; **Location:** country: Israel; locality: Herzliyya Hill; decimalLatitude: 32.15; decimalLongitude: 34.833333; **Event:** eventDate: 2007-04-12; **Record Level:** institutionCode: TAU
- **Type status:** Other material. **Occurrence:** catalogNumber: JSS50780; recordedBy: I. Yarom; individualCount: 1; sex: male; lifeStage: adult; **Taxon:** scientificName: *Eudorylasobliquus*; **Location:** country: Israel; locality: Holon; decimalLatitude: 32.0; decimalLongitude: 34.766667; **Event:** eventDate: 1995-03-23; **Record Level:** institutionCode: TAU
- **Type status:** Other material. **Occurrence:** catalogNumber: JSS50781; recordedBy: A. Freidberg; individualCount: 1; sex: male; lifeStage: adult; **Taxon:** scientificName: *Eudorylasobliquus*; **Location:** country: Israel; locality: Besor Nature Reserve; decimalLatitude: 31.3; decimalLongitude: 34.483333; **Event:** eventDate: 2005-05-11; **Record Level:** institutionCode: TAU
- **Type status:** Other material. **Occurrence:** catalogNumber: JSS50786; recordedBy: W. Kuslitzky; individualCount: 1; sex: male; lifeStage: adult; **Taxon:** scientificName: *Eudorylasobliquus*; **Location:** country: Israel; locality: Tel Aviv; decimalLatitude: 32.070472; decimalLongitude: 34.77425; **Event:** samplingProtocol: Malaise trap; eventDate: 2007-04-15; **Record Level:** institutionCode: TAU
- **Type status:** Other material. **Occurrence:** catalogNumber: JSS50787; recordedBy: W. Kuslitzky; individualCount: 1; sex: male; lifeStage: adult; **Taxon:** scientificName: *Eudorylasobliquus*; **Location:** country: Israel; locality: Tel Aviv; decimalLatitude: 32.070472; decimalLongitude: 34.77425; **Event:** samplingProtocol: Malaise trap; eventDate: 2007-04-15; **Record Level:** institutionCode: TAU
- **Type status:** Other material. **Occurrence:** catalogNumber: JSS50788; recordedBy: W. Kuslitzky; individualCount: 1; sex: male; lifeStage: adult; **Taxon:** scientificName: *Eudorylasobliquus*; **Location:** country: Israel; locality: Tel Aviv; decimalLatitude: 32.070472; decimalLongitude: 34.77425; **Event:** samplingProtocol: Malaise trap; eventDate: 2007-04-15; **Record Level:** institutionCode: TAU
- **Type status:** Other material. **Occurrence:** catalogNumber: JSS50789; recordedBy: A. Freidberg; individualCount: 1; sex: male; lifeStage: adult; **Taxon:** scientificName: *Eudorylasobliquus*; **Location:** country: Israel; locality: Bet Oren; decimalLatitude: 32.716667; decimalLongitude: 35; **Event:** eventDate: 2005-05-05; **Record Level:** institutionCode: TAU
- **Type status:** Other material. **Occurrence:** catalogNumber: JSS50790; recordedBy: A. Freidberg; individualCount: 1; sex: male; lifeStage: adult; **Taxon:** scientificName: *Eudorylasobliquus*; **Location:** country: Israel; locality: Bet Oren; decimalLatitude: 32.716667; decimalLongitude: 35.0; **Event:** eventDate: 2005-05-05; **Record Level:** institutionCode: TAU
- **Type status:** Other material. **Occurrence:** catalogNumber: JSS50792; recordedBy: A. Freidberg & F. Kaplan; individualCount: 1; sex: male; lifeStage: adult; **Taxon:** scientificName: *Eudorylasobliquus*; **Location:** country: Israel; locality: Park Rosh ha'Ayin; decimalLatitude: 32.083722; decimalLongitude: 34.955917; **Event:** eventDate: 1993-04-16; **Record Level:** institutionCode: TAU
- **Type status:** Other material. **Occurrence:** catalogNumber: JSS50797; recordedBy: W. Kuslitzky; individualCount: 1; sex: male; lifeStage: adult; **Taxon:** scientificName: *Eudorylasobliquus*; **Location:** country: Israel; locality: Tel Aviv; decimalLatitude: 32.070472; decimalLongitude: 34.77425; **Event:** samplingProtocol: Malaise trap; eventDate: 2007-04-15; **Record Level:** institutionCode: TAU
- **Type status:** Other material. **Occurrence:** catalogNumber: JSS50798; recordedBy: W. Kuslitzky; individualCount: 1; sex: male; lifeStage: adult; **Taxon:** scientificName: *Eudorylasobliquus*; **Location:** country: Israel; locality: Tel Aviv; decimalLatitude: 32.070472; decimalLongitude: 34.77425; **Event:** samplingProtocol: Malaise trap; eventDate: 2007-04-15; **Record Level:** institutionCode: TAU
- **Type status:** Other material. **Occurrence:** catalogNumber: JSS50799; recordedBy: W. Kuslitzky; individualCount: 1; sex: male; lifeStage: adult; **Taxon:** scientificName: *Eudorylasobliquus*; **Location:** country: Israel; locality: Tel Aviv; decimalLatitude: 32.070472; decimalLongitude: 34.77425; **Event:** samplingProtocol: Malaise trap; eventDate: 2007-04-20; **Record Level:** institutionCode: TAU
- **Type status:** Other material. **Occurrence:** catalogNumber: JSS50801; recordedBy: A.Freidberg; individualCount: 1; sex: male; lifeStage: adult; **Taxon:** scientificName: *Eudorylasobliquus*; **Location:** country: Israel; locality: Zomet Ha'Ela; decimalLatitude: 31.655833; decimalLongitude: 35.127444; **Event:** eventDate: 1999-04-04; **Record Level:** institutionCode: TAU
- **Type status:** Other material. **Occurrence:** catalogNumber: JSS50802; recordedBy: L. Friedman; individualCount: 1; sex: male; lifeStage: adult; **Taxon:** scientificName: *Eudorylasobliquus*; **Location:** country: Israel; locality: Zomet Ha'Ela; decimalLatitude: 31.655833; decimalLongitude: 35.127444; **Event:** eventDate: 2009-04-12; **Record Level:** institutionCode: TAU
- **Type status:** Other material. **Occurrence:** catalogNumber: JSS50805; recordedBy: A. Freidberg; individualCount: 1; sex: male; lifeStage: adult; **Taxon:** scientificName: *Eudorylasobliquus*; **Location:** country: Israel; locality: Zemah; decimalLatitude: 32.7; decimalLongitude: 35.583333; **Event:** eventDate: 2010-03-21; **Record Level:** institutionCode: CNC
- **Type status:** Other material. **Occurrence:** catalogNumber: JSS50807; recordedBy: L. Friedman; individualCount: 1; sex: male; lifeStage: adult; **Taxon:** scientificName: *Eudorylasobliquus*; **Location:** country: Israel; locality: Zomet Ha'Ela; decimalLatitude: 31.655833; decimalLongitude: 35.127444; **Event:** eventDate: 2009-04-12; **Record Level:** institutionCode: TAU
- **Type status:** Other material. **Occurrence:** catalogNumber: JSS50808; recordedBy: A. Freidberg; individualCount: 1; sex: male; lifeStage: adult; **Taxon:** scientificName: *Eudorylasobliquus*; **Location:** country: Israel; locality: Nahal Oren; decimalLatitude: 32.717361; decimalLongitude: 35.031417; **Event:** eventDate: 2005-05-03; **Record Level:** institutionCode: TAU
- **Type status:** Other material. **Occurrence:** catalogNumber: JSS50810; recordedBy: A. Freidberg; individualCount: 1; sex: male; lifeStage: adult; **Taxon:** scientificName: *Eudorylasobliquus*; **Location:** country: Israel; locality: Hof Rotem Shezaf; decimalLatitude: 32.766667; decimalLongitude: 35.633333; **Event:** eventDate: 2010-03-21; **Record Level:** institutionCode: TAU
- **Type status:** Other material. **Occurrence:** catalogNumber: JSS50811; recordedBy: A. Freidberg; individualCount: 1; sex: male; lifeStage: adult; **Taxon:** scientificName: *Eudorylasobliquus*; **Location:** country: Israel; locality: Hof Rotem Shezaf; decimalLatitude: 32.766667; decimalLongitude: 35.633333; **Event:** eventDate: 2010-03-21; **Record Level:** institutionCode: TAU
- **Type status:** Other material. **Occurrence:** catalogNumber: JSS50812; recordedBy: W. Kuslitzky; individualCount: 1; sex: male; lifeStage: adult; **Taxon:** scientificName: *Eudorylasobliquus*; **Location:** country: Israel; locality: Tel Aviv; decimalLatitude: 32.070472; decimalLongitude: 34.77425; **Event:** samplingProtocol: Malaise trap; eventDate: 2007-04-15; **Record Level:** institutionCode: TAU
- **Type status:** Other material. **Occurrence:** catalogNumber: JSS50813; recordedBy: W. Kuslitzky; individualCount: 1; sex: male; lifeStage: adult; **Taxon:** scientificName: *Eudorylasobliquus*; **Location:** country: Israel; locality: Tel Aviv; decimalLatitude: 32.070472; decimalLongitude: 34.77425; **Event:** samplingProtocol: Malaise trap; eventDate: 2007-04-15; **Record Level:** institutionCode: TAU
- **Type status:** Other material. **Occurrence:** catalogNumber: JSS50814; recordedBy: W. Kuslitzky; individualCount: 1; sex: male; lifeStage: adult; **Taxon:** scientificName: *Eudorylasobliquus*; **Location:** country: Israel; locality: Tel Aviv; decimalLatitude: 32.070472; decimalLongitude: 34.77425; **Event:** samplingProtocol: Malaise trap; eventDate: 2007-04-15; **Record Level:** institutionCode: TAU
- **Type status:** Other material. **Occurrence:** catalogNumber: JSS50821; recordedBy: A.Freidberg; individualCount: 1; sex: male; lifeStage: adult; **Taxon:** scientificName: *Eudorylasobliquus*; **Location:** country: Israel; locality: Zafririm; decimalLatitude: 31.65; decimalLongitude: 34.933333; **Event:** eventDate: 2002-03-30; **Record Level:** institutionCode: TAU
- **Type status:** Other material. **Occurrence:** catalogNumber: JSS50822; recordedBy: A. Freidberg; individualCount: 1; sex: male; lifeStage: adult; **Taxon:** scientificName: *Eudorylasobliquus*; **Location:** country: Israel; locality: Har Hermon; decimalLatitude: 33.3; decimalLongitude: 35.766667; **Event:** eventDate: 2000-05-17; **Record Level:** institutionCode: TAU
- **Type status:** Other material. **Occurrence:** catalogNumber: JSS50825; recordedBy: A. Freidberg; individualCount: 1; sex: male; lifeStage: adult; **Taxon:** scientificName: *Eudorylasobliquus*; **Location:** country: Israel; locality: Nahal Oren; decimalLatitude: 32.717361; decimalLongitude: 35.031417; **Event:** eventDate: 1998-05-30; **Record Level:** institutionCode: TAU
- **Type status:** Other material. **Occurrence:** catalogNumber: JSS50826; recordedBy: A. Freidberg; individualCount: 1; sex: male; lifeStage: adult; **Taxon:** scientificName: *Eudorylasobliquus*; **Location:** country: Israel; locality: Nahal Oren; decimalLatitude: 32.717361; decimalLongitude: 35.031417; **Event:** eventDate: 1998-05-30; **Record Level:** institutionCode: TAU
- **Type status:** Other material. **Occurrence:** catalogNumber: JSS52306; recordedBy: O. Ozden; individualCount: 1; sex: male; lifeStage: adult; associatedSequences: GB: MN549647; **Taxon:** scientificName: *Eudorylasobliquus*; **Location:** country: Cyprus; locality: Kyrenia; decimalLatitude: 35.3477; decimalLongitude: 33.1504; **Event:** samplingProtocol: Malaise trap; eventDate: 2018-03-20/27; **Record Level:** institutionCode: CNC
- **Type status:** Other material. **Occurrence:** catalogNumber: JSS50758; recordedBy: A. Freidberg; individualCount: 1; sex: male; lifeStage: adult; **Taxon:** scientificName: *Eudorylasobliquus*; **Location:** country: Israel; locality: Park haYarden; decimalLatitude: 32.9; decimalLongitude: 35.616667; **Event:** eventDate: 1999-04-14; **Record Level:** institutionCode: TAU
- **Type status:** Other material. **Occurrence:** catalogNumber: JSS50759; recordedBy: A. Freidberg; individualCount: 1; sex: male; lifeStage: adult; **Taxon:** scientificName: *Eudorylasobliquus*; **Location:** country: Israel; locality: Park haYarden; decimalLatitude: 32.9; decimalLongitude: 35.616667; **Event:** eventDate: 1999-04-14; **Record Level:** institutionCode: TAU
- **Type status:** Other material. **Occurrence:** catalogNumber: JSS50772; recordedBy: A. Freidberg; individualCount: 1; sex: male; lifeStage: adult; **Taxon:** scientificName: *Eudorylasobliquus*; **Location:** country: Israel; locality: Park haYarden; decimalLatitude: 32.9; decimalLongitude: 35.616667; **Event:** eventDate: 1999-04-14; **Record Level:** institutionCode: TAU
- **Type status:** Other material. **Occurrence:** catalogNumber: JSS50773; recordedBy: A. Freidberg; individualCount: 1; sex: male; lifeStage: adult; **Taxon:** scientificName: *Eudorylasobliquus*; **Location:** country: Israel; locality: Har Meron; decimalLatitude: 32.983333; decimalLongitude: 35.4; **Event:** eventDate: 1999-04-14; **Record Level:** institutionCode: TAU
- **Type status:** Other material. **Occurrence:** catalogNumber: JSS50809; recordedBy: L. Freidberg; individualCount: 1; sex: male; lifeStage: adult; **Taxon:** scientificName: *Eudorylasobliquus*; **Location:** country: Israel; locality: Nizzanim, A. Dunes; decimalLatitude: 31.718; decimalLongitude: 34.634639; **Event:** eventDate: 2009-04-06; **Record Level:** institutionCode: TAU
- **Type status:** Other material. **Occurrence:** catalogNumber: JSS50824; recordedBy: A. Freidberg; individualCount: 1; sex: male; lifeStage: adult; **Taxon:** scientificName: *Eudorylasobliquus*; **Location:** country: Israel; locality: Park haYarden; decimalLatitude: 32.9; decimalLongitude: 35.616667; **Event:** eventDate: 1997-05-31; **Record Level:** institutionCode: TAU
- **Type status:** Other material. **Occurrence:** catalogNumber: JSS50827; recordedBy: A. Freidberg; individualCount: 1; sex: male; lifeStage: adult; **Taxon:** scientificName: *Eudorylasobliquus*; **Location:** country: Israel; locality: Park haYarden; decimalLatitude: 32.9; decimalLongitude: 35.616667; **Event:** eventDate: 1997-05-07; **Record Level:** institutionCode: TAU
- **Type status:** Other material. **Occurrence:** catalogNumber: JSS50828; recordedBy: A. Freidberg; individualCount: 1; sex: male; lifeStage: adult; **Taxon:** scientificName: *Eudorylasobliquus*; **Location:** country: Israel; locality: Park haYarden; decimalLatitude: 32.9; decimalLongitude: 35.616667; **Event:** eventDate: 1997-05-07; **Record Level:** institutionCode: TAU
- **Type status:** Other material. **Occurrence:** catalogNumber: JSS50830; recordedBy: L. Friedman; individualCount: 1; sex: male; lifeStage: adult; **Taxon:** scientificName: *Eudorylasobliquus*; **Location:** country: Israel; locality: Park haYarden; decimalLatitude: 32.9; decimalLongitude: 35.616667; **Event:** eventDate: 1997-05-08; **Record Level:** institutionCode: TAU
- **Type status:** Other material. **Occurrence:** catalogNumber: JSS50833; recordedBy: A. Freidberg; individualCount: 1; sex: male; lifeStage: adult; **Taxon:** scientificName: *Eudorylasobliquus*; **Location:** country: Israel; locality: Park haYarden; decimalLatitude: 32.9; decimalLongitude: 35.616667; **Event:** eventDate: 1997-05-07; **Record Level:** institutionCode: TAU
- **Type status:** Other material. **Occurrence:** catalogNumber: 50782; recordedBy: A. Freidberg; individualCount: 1; sex: male; lifeStage: adult; **Taxon:** scientificName: *Eudorylasobliquus*; **Location:** country: Israel; locality: Besor Nature Reserve; decimalLatitude: 31.3; decimalLongitude: 34.483333; **Event:** eventDate: 2005-05-11; **Record Level:** institutionCode: TAU

#### Diagnosis

**Figure 19.**
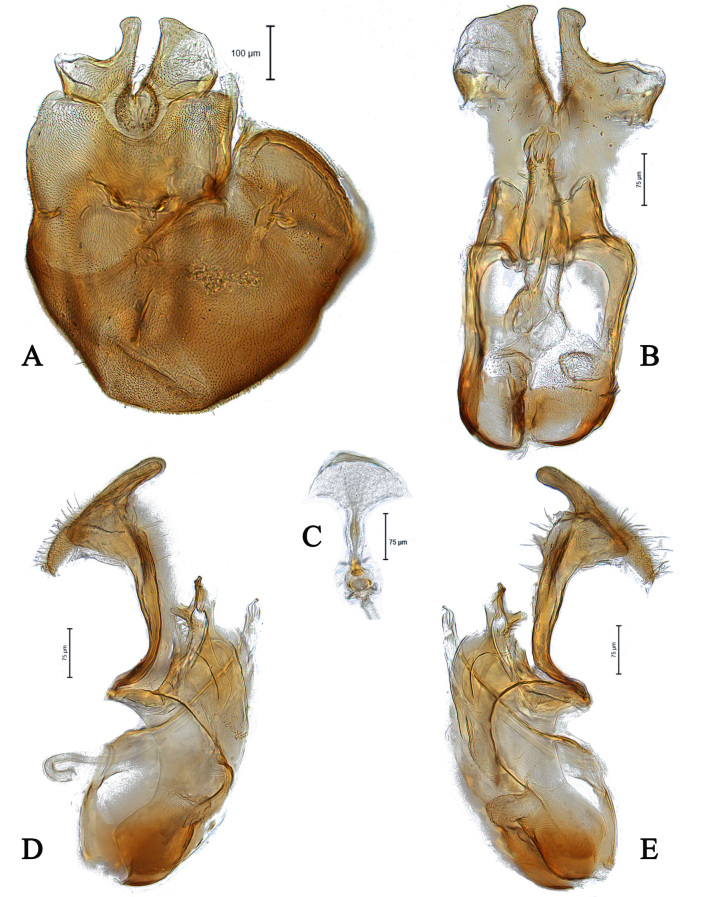
Male genitalia of *Eudorylasobliquus* (JSS50762) (A) in dorsal view; (B) in ventral view; (C) ejaculatory apodeme; (D, E) in lateral view.

This species can be recognised by the asymmetrical surstyli, base of the right surstylus rectangular shape with inner finger-like projection in dorsal view (Fig. 19A); epandrium wider than long (Fig. 19A); phallic guide dorsally with two finger-like projections situated half way up in lateral view (Fig. 19D, E); small and equal gonopods in ventral view (Fig. 19B). The genitalia of this species are similar to *E.jenkinsoni* Coe, 1966. It differs by smaller size; shorter setae on the abdominal tergite 2-5; right surstylus longer than wide in dorsal view (Fig. 19A).

#### Distribution

Belgium, Bulgaria, Cyprus, Czech Republic, France, Germany, England, Greece, Hungary, Ireland, Israel, Italy, Netherlands, Portugal, Slovakia, Spain, Switzerland, Turkey (Kehlmaier 2005a, Skevington 2020) (Fig. 12).

#### Notes

DNA barcodes of *Eudorylasobliquus* overlap with *E.jenkinsoni*. See the notes under *E.jenkinsoni* for more details.

### Eudorylas pannonicus

(Becker, 1897)

1FA0B4B1-F50E-5F0B-BDFF-E988F499E892

Pipunculus pannonicus Becker 1897:51

#### Materials

- **Type status:** Other material. **Occurrence:** catalogNumber: JSS52207; recordedBy: E. Gilasian; individualCount: 1; sex: male; lifeStage: adult; associatedSequences: GB: MN549657; **Taxon:** scientificName: *Eudorylaspannonicus*; **Location:** country: Iran; stateProvince: Khuzestan; locality: Shush; decimalLatitude: 32.1; decimalLongitude: 48.43; **Event:** samplingProtocol: Malaise trap; eventDate: 2015-08-20; **Record Level:** institutionCode: CNC
- **Type status:** Other material. **Occurrence:** catalogNumber: JSS52305; recordedBy: O. Ozden; individualCount: 1; sex: male; lifeStage: adult; associatedSequences: GB: MN549656; **Taxon:** scientificName: *Eudorylaspannonicus*; **Location:** country: Cyprus; locality: Kyrenia; decimalLatitude: 35.347; decimalLongitude: 33.150400; **Event:** samplingProtocol: Malaise trap; eventDate: 2017-11-05/12; **Record Level:** institutionCode: CNC

#### Diagnosis

This species can be recognised by the shape of surstyli in dorsal view, base of both surstyli slightly rectangular-shaped, right surstylus with long inner finger-like projection curved to left surstylus in dorsal view (Fig. 18A); gonopods unequal, right higher than left one, left one with two small projections in ventral view (Fig. 18B); phallic guide straight with apical projection pointing upwards in lateral view (Fig. 18C).

#### Distribution

Bulgaria, Croatia, Cyprus, France, Greece, Hungary, Iran, Italy, Romania (Skevington 2002b, Kehlmaier 2005a, Gharali et al. 2008) (Fig. 6).

#### Notes

Based on the shape of the genitalia, this species belongs to *E.pannonicus* form A (see Kehlmaier 2005a Fig. 63a). DNA barcodes of *Eudorylaspannonicus* and *E.nasicus* sp. n. are very similar (1.19% pairwise divergence). The genitalia of these species differ by the shape of right surstylus and phallic guide. The finger-like projection of the right surstylus is longer and the projection of the phallic guide is straighter in *E.pannonicus*. The differences are small, but we and C. Kehlmaier (pers. comm.) feel that they are different species and have treated them as such.

### Eudorylas zermattensis

(Becker, 1897)

66E064F6-0763-58A7-BAE8-35599A0B30DB

Pipunculus zermattensis Becker, 1897: 77.

#### Materials

- **Type status:** Other material. **Occurrence:** catalogNumber: JSS52157; recordedBy: M. Zardouei; individualCount: 1; sex: male; lifeStage: adult; **Taxon:** scientificName: *Eudorylaszermattensis*; **Location:** country: Iran; stateProvince: Kermanshah; locality: Sarpolezahab; decimalLatitude: 34.466667; decimalLongitude: 45.816667; **Event:** samplingProtocol: Malaise trap; eventDate: 2016-09-07; **Record Level:** institutionCode: CNC
- **Type status:** Other material. **Occurrence:** catalogNumber: JSS52158; recordedBy: M. Zardouei; individualCount: 1; sex: male; lifeStage: adult; **Taxon:** scientificName: *Eudorylaszermattensis*; **Location:** country: Iran; stateProvince: Kermanshah; locality: Sarpolezahab; decimalLatitude: 34.466667; decimalLongitude: 45.816667; **Event:** samplingProtocol: Malaise trap; eventDate: 2016-09-07; **Record Level:** institutionCode: CNC
- **Type status:** Other material. **Occurrence:** catalogNumber: JSS52162; recordedBy: M. Zardouei; individualCount: 1; sex: male; lifeStage: adult; associatedSequences: GB: MN549662; **Taxon:** scientificName: *Eudorylaszermattensis*; **Location:** country: Iran; stateProvince: Kermanshah; locality: Sarpolezahab; decimalLatitude: 34.466667; decimalLongitude: 45.816667; **Event:** samplingProtocol: Malaise trap; eventDate: 2016-09-07; **Record Level:** institutionCode: CNC
- **Type status:** Other material. **Occurrence:** catalogNumber: JSS52210; recordedBy: E. Gilasian; individualCount: 1; sex: male; lifeStage: adult; **Taxon:** scientificName: *Eudorylaszermattensis*; **Location:** country: Iran; stateProvince: Tehran; locality: Tehran; decimalLatitude: 35.783333; decimalLongitude: 51.4; **Event:** samplingProtocol: Malaise trap; eventDate: 2010-05-01; **Record Level:** institutionCode: CNC
- **Type status:** Other material. **Occurrence:** catalogNumber: JSS52159; recordedBy: M. Zardouei; individualCount: 1; sex: male; lifeStage: adult; **Taxon:** scientificName: *Eudorylaszermattensis*; **Location:** country: Iran; stateProvince: Kermanshah; locality: Sarpolezahab; decimalLatitude: 34.466667; decimalLongitude: 45.816667; **Event:** samplingProtocol: Malaise trap; eventDate: 2016-09-07; **Record Level:** institutionCode: CNC

#### Diagnosis

**Figure 20.**
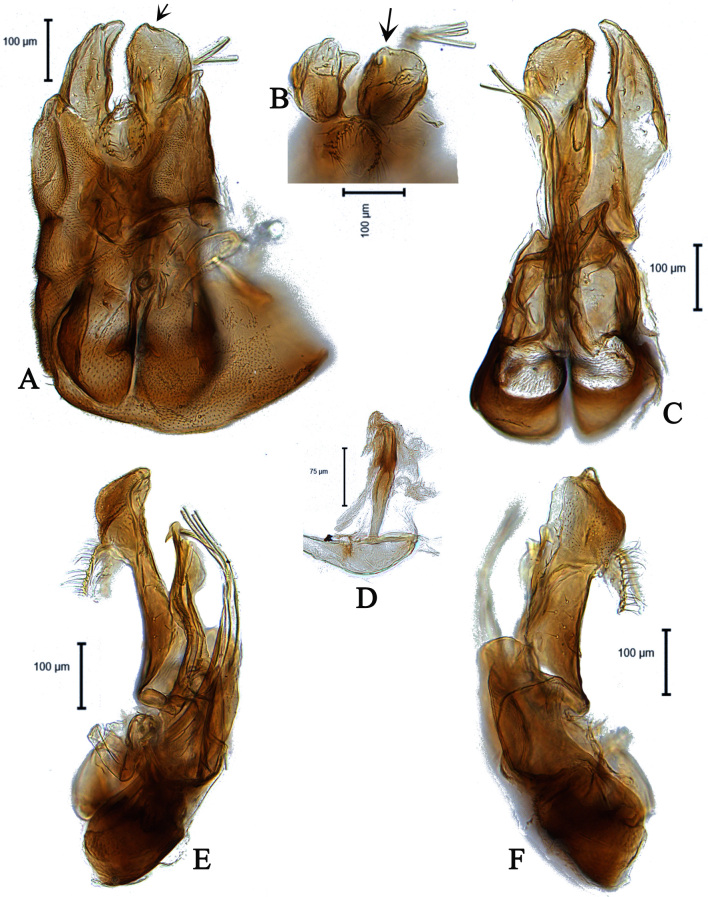
Male genitalia of *Eudorylaszermattensis* (JSS52162) (A) in dorsal view; (B) surstyli in dorsal view; (C) in ventral view; (D) ejaculatory apodeme; (E, F) in lateral view.

This species can be recognised by ovate surstyli in ground shape, longer than wide in dorsal view (Fig. 20B), right surstylus with small projection towards left surstylus (Fig. 20B); gonopods unequal, right higher than left one in ventral view (Fig. 20C); phallic guide medially with small bulge and apically with hook-like projection towards subepandrial sclerite in lateral view (Fig. 20E).

#### Distribution

Andorra, Austria, Belgium, Czech Republic, Denmark, England, Finland, France, Germany, Greece, Hungary, Iran, Israel, Italy, Latvia, Netherlands, Portugal, Romania, Slovakia, Spain, Sweden, Turkey, Uzbekistan, former Yugoslavia (Kehlmaier 2005a, Kehlmaier et al. 2019, Skevington 2020) (Fig. 12).

#### Notes

Intraspecific distances of *E.zermattensis* specimens range from 0.0-0.71%.

## Identification Keys

### Key to males of *Eudorylas* species in the Middle East

**Table:** 

1	Phallic guide divided (Fig. 7C, Fig. 10E)	2
–	Phallic guide not divided	3
2	Right phallic guide with distinct projections in ventral view (Fig. 10C)	*E.corniculans* Motamedinia & Skevington sp. n.
–	Right phallic guide without branch in ventral view (Fig. 7C)	*E.bipertitus*
3	Phallic guide straight in lateral view (Fig. 8D,Fig. 13D,Fig. 14D)	4
–	Phallic guide not straight in lateral view (Fig. 3D, Fig. 20E)	15
4	Phallic guide with distinct projection dorsomedially (Fig. 8B, Fig. 14B)	5
–	Phallic guide without distinct projection dorsomedially	12
5	Phallic guide with one dorsal or dorsolateral projection (Fig. 8B, Fig. 14B)	6
–	Phallic guide with two dorsal or dorsolateral projections	9
6	Phallic guide with projection pointing downwards in ventral view (Fig. 13B, Fig. 14B)	7
–	Phallic guide with projection pointing upwards in ventral view (Fig. 8B)	8
7	Left surstylus with a triangular projection in basal half in dorsal view (Fig. 13A); right gonopod with a distinct finger-like projection in ventral view (Fig. 13B)	*E.flavicrus*
–	Left surstylus without a triangular projection in basal half in dorsal view (Fig. 14A); right gonopod without a distinct finger-like projection in ventral view (Fig. 14B)	*E.fluviatilis*
8	Base of left surstylus as long as wide in dorsal view (Fig. 8A); left gonopod with one small projection in ventral view (Fig. 8B)	*E.blascoi*
–	Base of left surstylus longer than wide in dorsal view (Fig. 18A); left gonopod with two small projections in ventral view (Fig. 18B)	*E.pannonicus*
9	Gonopods unequal in height in ventral view (Fig. 5B, Fig. 17B)	10
–	Gonopods equal in height in ventral view (Fig. 19B)	11
10	Surstyli longer than wide; right surstylus with a finger-like projection on inner side in dorsal view (Fig. 17A)	*E.nasicus* Motamedinia & Skevington sp. n.
–	Surstyli wider than long; both surstyli with a finger-like projection on inner side in dorsal view (Fig. 5A)	*E.bihamatus* Motamedinia & Skevington sp. n.
11	Right surstylus longer than wide in dorsal view (Fig. 19A)	*E.obliquus*
–	Right surstylus wider than long in dorsal view; (Kehlmaier 2005a: Figure 31a)	*E.jenkinsoni*
12	Base of phallic guide broadened (Kehlmaier 2005a: Figure 50b)	*E.longifrons*
–	Base of phallic guide not broadened	13
13	Phallus coiled twice (Kehlmaier 2005a: Figure 47g)	*E.auctus*
–	Phallus coiled once	14
14	Gonopods equal in height (Fig. 15B)	*E.gemellus*
–	Left gonopod higher than right one (Kehlmaier 2005a: Figure 37a)	*E.fuscipes*
15	Base of right surstylus as long as wide (Kehlmaier 2005a: Figure 69j)	*E.chvalai*
–	Base of right surstylus not as long as wide	16
16	Right surstylus wider than long (Kehlmaier 2005a: Figure 30e)	*E.fascipes*
–	Right surstylus longer than wide	17
17	Left surstylus slightly rounded, without distinct projection in dorsal view (Fig. 20A, Fig. 20B)	*E.zermattensis*
–	Left surstylus not rounded, with distinct projection in dorsal view (Fig. 3A)	*E.avis* Motamedinia & Skevington sp. n.

## Discussion

### DNA barcoding

Based on morphology and DNA barcoding, the present paper introduces four new species of *Eudorylas*, *E.avis* sp. n., *E.bihamatus* sp. n., *E.corniculans* sp. n. and *E.nasicus* sp. n. and associates or confirms the association of males and females of three species: *E.blascoi, E.corniculans* sp. n. and *E.fluviatilis.* DNA sequence data are provided for 11 Middle Eastern *Eudorylas* species.

Interspecific genetic distances within the Middle Eastern *Eudorylas* range from 1.3% (*E.nasicus* sp. n. to *E.pannonicus*) to 16.2% (*E.obliquus* to *E.bihamatus* sp. n.), while intraspecific genetic distances range from 0% (within both *E.blascoi* and *E.fluviatilis*) to 1.7% (within both *E.obliquus* and *E.fluviatilis*). Based on uncorrected pairwise genetic distances (p-distance), *E.avis* sp. n. is close to *E.fluviatilis* (6.4%) and *E.gemellus* (6.7%), while *E.corniculans* sp. n. is close to *E.bihamatus* sp. n. (5.2%). *Eudorylasnasicus* sp. n. is most similar to *E.pannonicus*, differing by 1.2% (Suppl. material 1).

## Supplementary Material

XML Treatment for Eudorylas

XML Treatment for Eudorylas auctus

XML Treatment for Eudorylas avis

XML Treatment for Eudorylas bihamatus

XML Treatment for Eudorylas bipertitus

XML Treatment for Eudorylas blascoi

XML Treatment for Eudorylas chvalai

XML Treatment for Eudorylas corniculans

XML Treatment for Eudorylas fascipes

XML Treatment for Eudorylas flavicrus

XML Treatment for Eudorylas fuscipes

XML Treatment for Eudorylas fluviatilis

XML Treatment for Eudorylas gemellus

XML Treatment for Eudorylas jenkinsoni

XML Treatment for Eudorylas longifrons

XML Treatment for Eudorylas nasicus

XML Treatment for Eudorylas obliquus

XML Treatment for Eudorylas pannonicus

XML Treatment for Eudorylas zermattensis

0968D494-DD8C-5447-BE7E-BE4AD6DDFFCE 10.3897/BDJ.8.e53609.suppl1 Supplementary material 1 Uncorrected pairwise distances amongst *Eudorylas* species in the Middle East. Data type genetic distances File: oo_425542.xlsx https://binary.pensoft.net/file/425542 Behnam Motamedinia, Jeff Skevington, Scott Kelso

## Figures and Tables

**Table 1.** Cytochrome c oxidase subunit I mitochondrial gene primers.

Gene name/region	Forward primer name	Forward primer sequence (5'-3')	Primer reference	Reverse primer name	Reverse primer sequence (5'-3')	Primer reference
COI Barcode	LCO1490	GGTCAACA AATCATAAA GATATTGG	Folmer et al. 1994	COI-Dipt-2183R	CCAAAAAATC ARAATARRTG YTG	Gibson et al. 2011
COI-Fx-A (5' end of barcode)	LCO1490	GGTCAACA AATCATAAA GATATTGG	Folmer et al. 1994	COI-SYR-1762R	CGDGGRAAD GCYATRTCDGG	Motamedinia et al. 2019
COI-Fx-B (middle of barcode)	COI-SYR-342F	GGDKCHCC NGAYATRGC	Motamedinia et al. 2019	COI-SYR-1976R	GWAATRAART TWACDGCHCC	Motamedinia et al. 2019
COI-Fx-C (3' end of barcode)	COI-SYR-1957F	GGDATWTC HTCHATYYTAGG	Motamedinia et al. 2019	COI-Dipt-2183R	CCAAAAAATCA RAATARRTGYTG	Gibson et al. 2011
